# Janus USPION modular platform (JUMP) for theranostic ultrasound-mediated targeted intratumoral microvascular imaging and DNA/miRNA delivery

**DOI:** 10.7150/thno.78454

**Published:** 2022-11-07

**Authors:** Ragnhild D. Whitaker, Julius L. Decano, Catherine Gormley, Carl A. Beigie, Cari Meisel, Glaiza A. Tan, Ann-Marie Moran, Nicholas J. Giordano, Yoonjee Park, Peng Huang, Sean Andersson, Donald Gantz, Aaron K. Grant, Nelson Ruiz-Opazo, Victoria L.M. Herrera, Joyce Y. Wong

**Affiliations:** 1 Department of Biomedical Engineering, Boston University, Boston, MA, USA.; 2 Whitaker Cardiovascular Institute, Boston University, Boston, MA, USA.; 3 Department of Medicine, Boston University School of Medicine, Boston, MA, USA.; 4 Department of Mechanical Engineering, Boston University, Boston, MA, USA.; 5 Division of Systems Engineering, Boston University, Boston, MA, USA.; 6 Department of Physiology and Biophysics, Boston University, Boston, MA, USA.; 7 Beth Israel Deaconess Medical Center, Harvard Medical School, Boston, MA, USA.; 8 Division of Materials Science and Engineering, Boston University, Boston, MA, USA.

**Keywords:** Nanomedicine, Janus nanoparticle, pancreatic cancer, nucleic acid delivery, USPION, nano-micro hybrid platform, modular nanotheranostics

## Abstract

**Rationale:** High mortality in pancreatic cancer (PDAC) and triple negative breast cancer (TNBC) highlight the need to capitalize on nanoscale-design advantages for multifunctional diagnostics and therapies. DNA/RNA-therapies can provide potential breakthroughs, however, to date, there is no FDA-approved systemic delivery system to solid tumors.

**Methods:** Here, we report a Janus-nanoparticle (jNP)-system with modular targeting, payload-delivery, and targeted-imaging capabilities. Our jNP-system consists of 10 nm ultrasmall superparamagnetic iron oxide nanoparticles (USPION) with opposing antibody-targeting and DNA/RNA payload-protecting faces, directionally self-assembled with commercially available zwitterionic microbubbles (MBs) and DNA/RNA payloads.

**Results:** Sonoporation of targeted jNP-payload-MBs delivers functional reporter-DNA imparting tumor-fluorescence, and micro-RNA126 reducing non-druggable KRAS in PDAC-Panc1 and TNBC-MB231 xenografted tumors. The targeting jNP-system enhances ultrasound-imaging of intra-tumoral microvasculature using less MBs/body weight (BW). The jNP-design enhances USPION's T2*-magnetic resonance (MR) and MR-imaging of PDAC-peritoneal metastases using less Fe/BW.

**Conclusion:** Altogether, data advance the asymmetric jNP-design as a potential theranostic Janus-USPION Modular Platform - a JUMP forward.

## Introduction

Nucleic acid delivery to tumors such as pancreatic cancer 1 and triple negative breast cancer 2 remains a challenge due to high mortality and resistance. Nucleic acids are rapidly degraded in the circulation, and current systemic nano-delivery platforms exhibit clinical limitations in safety and efficacy in disease states. These range from immunological activation with adverse cytokine storms 3, minimal endosomal escape of nucleic acid cargo (< 2%) 4, and/or exocytosis of a majority (> 70%) of internalized inhibitory RNA (RNAi)-payloads 5,6. Ultrasound-mediated nucleic acid delivery can bypass endosomal uptake limitations by producing pores in cell membranes through sonoporation (reviewed in 7), but has not yet translated to the clinic due to short (~ 5 min) microbubble circulation half-lives and insufficient payload delivery 8 - even when using high payload cationic microbubbles (CMBs) 9 due to charge-associated cytotoxicity 10. Moreover, it may be difficult to achieve efficient gene expression, which requires dissociation between plasmid DNA and the microbubble cationic lipids 8.

Cationic polyethyleneimine (PEI) has been studied extensively as a nucleic acid carrier and has been used in conjunction with ultrasound and magnetic resonance contrast agents in theranostic platforms 9, 11. Still, PEI exhibits charge-associated cytotoxicity 12, and efforts to improve its safety profile include covalently modifying PEI with polyethyleneglycol (PEG) and other polymers (e.g. 11 and reviewed in 13, 14); reducing the amount of PEI used 15; and substituting linear for branched PEI 16. Developing effective nano/microparticle theranostic platforms remains challenging: incorporating drug and nucleic acid payloads and targeting moieties into/onto ultrasound and magnetic resonance contrast agents can compromise diagnostic and therapeutic functions due to competing structure-function relationships 17. Moreover, the structural organization of the various components in a theranostic agent is important to achieve effective nucleic acid delivery. For example, PEI-coated SPIONs aggregate in the presence of DNA 18, hence unsafe for systemic clinical applications and ineffective for DNA/RNA payload release.

To overcome these key limitations and to avoid lethal adverse events, nanoparticle (NP) design must consider charge, hydrophilicity, shape, size 19, structural stability, and biocompatibility. The protein corona acquired by NPs in circulation must be minimized to preserve NP functionality, as well as to prevent neoepitopes triggering hyperinflammatory cytokine storms 20, 21. While focus has been on enhancing endosomal escape and minimizing exocytosis to overcome inefficient endocytic uptake, NPs attaining endocytosis-independent delivery of [DNA/RNAi]-payloads provide a promising alternative approach 22. Equally important, modular multifunctionalities 23 accommodating multiple targeting and payload moieties are needed to address dynamic temporal and spatial genetic/epigenetic heterogeneity characteristics in most cancers.

Janus NPs spatially separate multifunctional components to improve functionalities 23, but to date, biological applications of Janus NPs have been limited. There are no FDA-approved Janus NPs for nucleic acid delivery systems 6, 24, 25. This is partly due to protein coronas adsorbed *in vivo* leading to loss of NP function and/or increased toxicity 3. We therefore tested the hypothesis that a Janus NP prepared by unidirectional covalent layering on a tiered PEG-brush can provide a stable nano-delivery system with modular theranostic multifunctionalities, *in vivo* biocompatibility, and an immunoglobulin targeting face that provides a pre-defined protein corona and avoids unpredictable protein-corona adsorption. Importantly, spatial segregation of the targeting and payload functions on a Janus NP reduces the amount of material used.

Here we present a covalently layered Janus nanoparticle, jNP, with a 10 nm USPION core, tiered PEG brush, antibody targeting face and an opposing cationic carrier face. We selected linear-PEI (25 kDa) for the tiered-PEG cationic face based on studies reporting absence of acute adverse events for PEI-[DNA/siRNA] nano-polyplexes 26, 27 during a 2-week observation period. We find - without cytokine elevation or adverse events - jNPs exhibit modular theranostic capabilities with high efficiency: i) jNP-microbubble(MB)-targeted delivery of functional DNA/miRNA to xenograft tumors in an endocytosis-independent manner via operator-controlled MB sonoporation, and ii) modular imaging: enhanced ultrasound molecular imaging of intratumoral vasculature (jNP-MBs) and enhanced T2* MR imaging of tumor-selective enhanced permeation retention (EPR) effects (jNPs). Altogether, data provide proof-of-principle for this jNP-design as a modular dual imaging and DNA/miRNA systemic delivery system for cancer, thus advancing the theranostic potential of Janus USPIONs Modular Platform - JUMP - for further study.

## Results

### Janus nanoparticles (jNPs)

**Figure 1:**
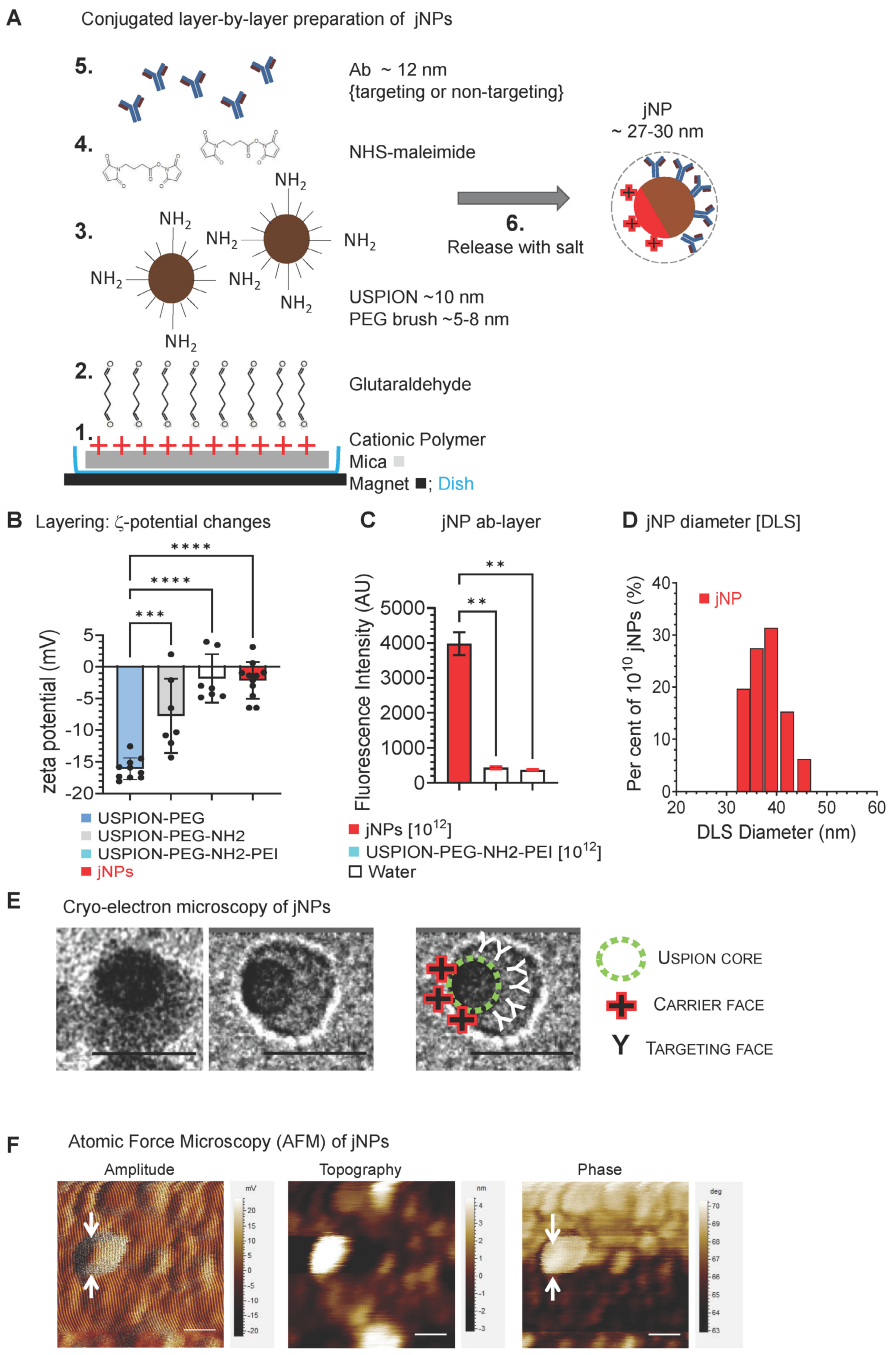
** Conjugated layer-by-layer preparation and cryo-TEM/AFM imaging of Janus nanoparticles (jNPs).** (**A**) Schematic diagram of directional layer-by-layer method to prepare jNPs. See Methods for detailed procedure. Layers are: **1**: cationic polymer, PEI, adsorbed onto a mica sheet; **2**: glutaraldehyde, conjugated to amines in the PEI layer; **3**: Fe_3_O_4_ ~ 10 nm USPION with mixed partially amine-terminated PEG_2K/3.4K_ brush (~ 5-8 nm) conjugated to glutaraldehyde layer; **4**: maleimide layer from conversion of free amines on USPION core by N-hydroxy-succinimide maleimide (NHS-maleimide); **5**: targeting antibodies (Ab) conjugated to layer-3 via NHS-maleimide linker (layer-4); **6**: asymmetrically functionalized jNPs released from mica sheet with salt. (**B**) Differential zeta potential levels of partial jNP layered-composition stages with ultrasmall 10 nm SPION cores: uspion-peg, uspion-peg-nh_2_, uspion-peg-nh_2_-pei, and jNP; mean ± s.d., n = 7-11 replicates/group, each replicate = ~ 10^10^ NPs, three independent experiments, ANOVA * *p* < 0.05; ** *p*<0.01. (**C**) Fluorescence intensity levels, documenting conjugation of AF594-labeled antibodies (Ab) as final layer (jNP) and targeting-face of jNPs compared to uspion-peg-nh_2_-pei and water controls (mean ± s.d. of DLS readings from 10^12^ jNPs and USPION-PEG-NH_2_-PEI nanoparticles). ANOVA * *p* < 0.01. (**D**) Representative frequency plot of % of 10^12^ jNPs at specified hydrodynamic diameters (nm) obtained via dynamic light scattering (DLS) at time-0. (**E**) Representative cryo-TEM images of jNPs from two independent jNP preparations showing an asymmetric ~ 22-32 nm particle with an electro-dense USPION core closer to the PEI cationic carrier-face and targeting antibodies (~ 12 nm) comprising the opposing targeting-face. Scale bar = 20 nm. (**F**) Representative atomic force microscopy (AFM) multi-parameter images (amplitude, topography, and phase) of individual jNPs. Scale bar = 50 nm.

Janus-nanoparticles (jNPs) are prepared using a modified surface-initiated conjugation method. Layer-1 [“payload-face”, linear polyethyleneimine 25 kDa (LPEI_25K_)] adsorbs onto mica via electrostatic interactions (**Figure 1A**). Layer-2 (glutaraldehyde) covalently links amines in LPEI_25K_ (Layer-1) with amine-terminated PEG chains in Layer-3 (USPIONs pre-synthesized with a tiered polymer brush) 28, 29. The mixed PEG_2K/3.4K-NH2_ brush prevents USPION aggregation 29, 30 and exposes free amines for conjugation of targeting-antibody 31 (**Figure 1A**). The 10 nm USPION-size is optimal for MR-imaging 32 which can be further enhanced by antifouling PEG-coating 33. Layer-4 (N-hydroxysuccinimide-ester-maleimide [NHS-MAL]) 34 crosslinks free amines on Layer-3 to cysteine thiol groups of targeting-antibodies, Layer-5 “targeting-face”. Zeta potential (**Figure 1B**) and fluorescence (**Figure 1C**) confirm layer-specific components at different stages of jNP assembly with approximately 3-12 antibodies/jNP and ~ 10^12^-10^13^ jNPs/mica with average hydrodynamic diameters ~ 30-50 nm (**Figure 1D**).

Cryo-transmission electron microscopy (cryo-TEM) images show jNPs are largely asymmetric 22-30 nm particles with off-center USPION-core and ~ 12 nm opposing tapered face (**Figure 1E**). Atomic force microscopy (AFM) confirms asymmetrically functionalized USPION cores, distinguishing dense USPION cores from less dense material observed in amplitude and phase AFM-images (**Figure 1F**, **Figure S1-A**): AFM-imaging shows ~ 55 nm - larger than cryo-TEM measurements, likely due to flattening during AFM-imaging.

### jNP Baseline characterization

**Figure 2:**
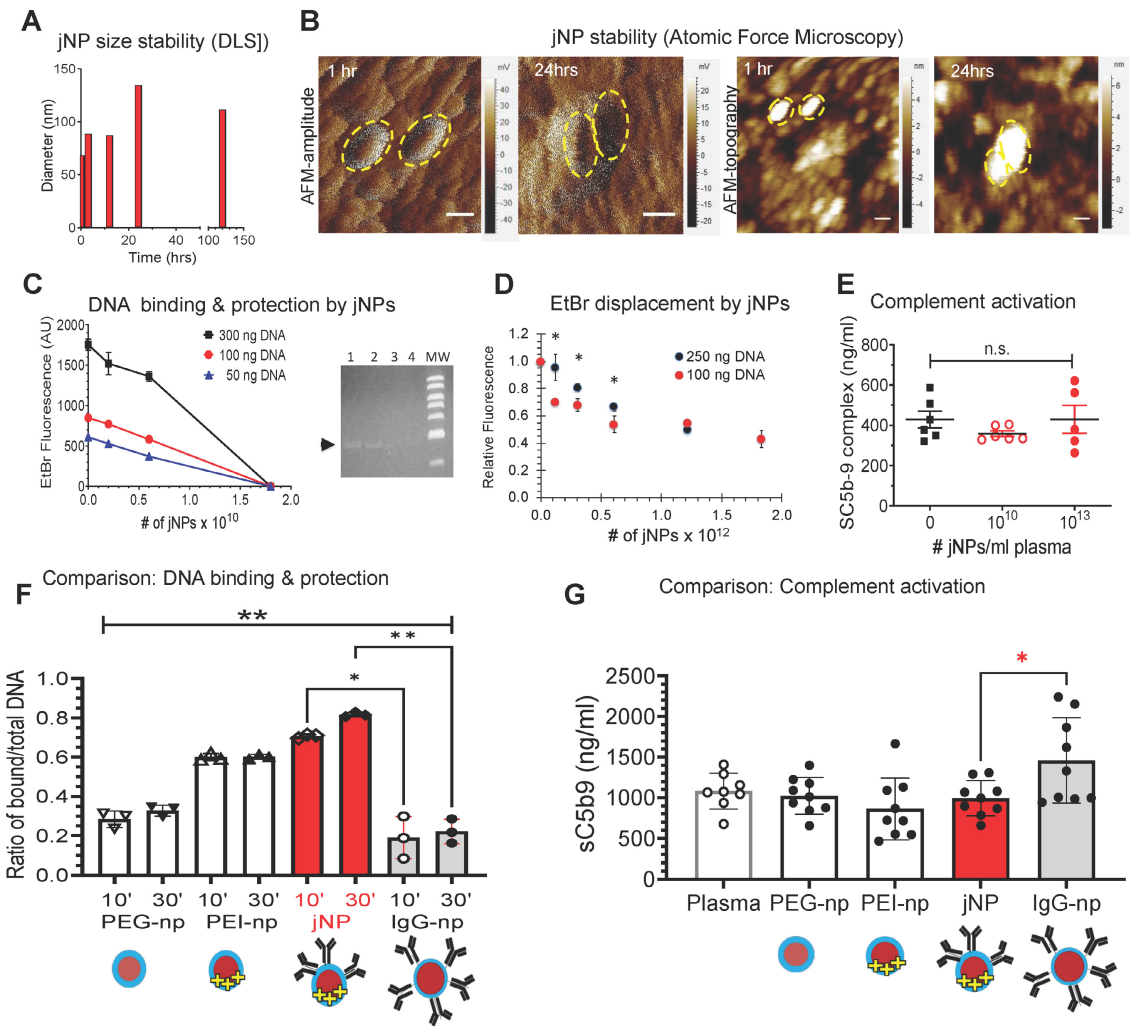
***In vitro* characterization of jNPs. (A)** Representative serum-stability time-course plot of hydrodynamic diameters (nm) of jNPs from 0-120 h in 75% serum, one way ANOVA *P* > 0.63. **(B)** Representative AFM amplitude and topography images taken of jNPs at t-1 h and t-24 h in 85% serum. Scale bar = 50 nm. **(C)** Ethidium bromide (EtBr) dye exclusion assay for different amounts of DNA (300, 100, and 50 ng RFP-minigene plasmid DNA) exposed to increasing amounts of jNPs (# of jNPs x 10^10^) for 10 min incubation. Level of free DNA available for EtBr intercalation defines unbound or unprotected by jNPs, is indicated by level of EtBr fluorescence intensity (arbitrary units, AU) emitted from EtBr upon intercalation into free DNA. Fluorescence (Ex. 260/Em.590 nm) at 10 min done in duplicate; highest jNP point in triplicate. Agarose gel analysis of EtBr fluorescence after intercalation into 'free' RFP plasmid 4.7 kb DNA (black arrow). Lanes 1-4, increasing (0, 0.2, 0.6, 1.8 x 10^10^) jNPs added to 100 ng RFP-DNA for 10 min. MW, bands from 10, 8, 6, 5, 4, 3, and 2 kb DNA markers. **(D)** Graph of jNP dose-dependent displacement of fluorescent DNA-intercalated EtBr. Two amounts of DNA were tested: 100 ng, and 250 ng. jNPs from 0 - 1.75 x 10^12^ nps were used to test dose-dependent displacement of EtBr by jNPs. **(E)** Graph of *in vitro* testing of jNPs triggering complement activation. Using amounts that span levels of jNPs used *in vivo* ~ 10^11^ jNPs/mL plasma, jNPs at 10^10^/mL up to 10^13^ jNPs/mL were tested for complement activation by measuring levels of terminal complex SC5b-9 compared to human plasma control with no jNPs. **(F)** Comparison of DNA-binding and protection from EtBr intercalation by jNP compared with pertinent controls: PEG-np (PEG-SPION core), PEI-np (PEG-np with conjugated PEI-face), IgG-np (isotype IgG antibody layer surrounds PEG-SPION core, no conjugated PEI-face) at 2 time points: 10 min (10'), 30 min (30'). Diagrams depict respective NP design. Data presented as mean ± s.d., Kruskal Wallis with Dunn's multiple comparison pairwise testing: (*, p < 0.05; **, p < 0.001), 300 ng DNA/10^10^ jNPs, n = 3 independent experiments. **(G)** Comparison of complement activation *in vitro* of jNPs compared with pertinent controls: human normal plasma with no NPs, PEG-np, PEI-np, and IgG-np identical to that used in Figure 2F. One-way ANOVA with Dunnett's multiple pair-wise comparison for jNP and IgG-np: *, *p* < 0.05, n = 8-9/group, 5 groups, each group with 10^12^ nanoparticles.

jNP size and morphology in 75% serum do not exhibit significant size changes in the first 12 h (**Figure 2A**). Although batch-to-batch variations occur, size increases are limited (**Figure 2A**). Likewise, AFM amplitude and topography images show jNPs retain characteristic asymmetric morphologies seen on cryo-TEM for at least 24 h in 75% serum (**Figure 2B**), indicating stability required for *in vivo* use.

To demonstrate jNP nucleic acid carrier function, jNP binding to lambda phage double-stranded DNA is visualized by AFM (**Figure S1-B**), and jNP-DNA binding is confirmed via ethidium bromide (EtBr) dye exclusion assay 35,36. jNPs bind DNA in a concentration-dependent manner and prevent EtBr-intercalation into DNA base-pairs as detected by decreasing EtBr-fluorescence with increasing number of jNPs (**Figure 2C**). Agarose gel electrophoresis confirms jNP-dependent binding to DNA, and resultant protection of DNA from EtBr intercalation (**Figure 2C-right panel**). Moreover, the reverse-order binding experiment shows jNPs displace EtBr already intercalated into DNA (4:1 DNA-EtBr ratio) (**Figure 2D**), indicating higher affinity and stability of jNP binding to DNA compared to EtBr. Additionally, jNPs at 10^10^ and 10^13^ jNPs/mL plasma concentrations do not induce complement activation (**Figure 2E**).

To demonstrate advantages of the Janus-design, comparative analyses of jNPs with non-Janus NP-constructs - USPIONs with isotropic PEG (PEG-np), PEG-PEI (PEI-np), PEG-IgG (IgG-np) - show that jNPs have the highest amount of bound-DNA protected from EtBr intercalation (**Figure 2F**). Importantly, jNPs do not induce complement activation in contrast to isotropic IgG-np (**Figure 2G**). These observations support advantages of the jNP-design in segregating targeting- and payload-functionalities.

### Assembly of jNP-DNA-MBs

**Figure 3:**
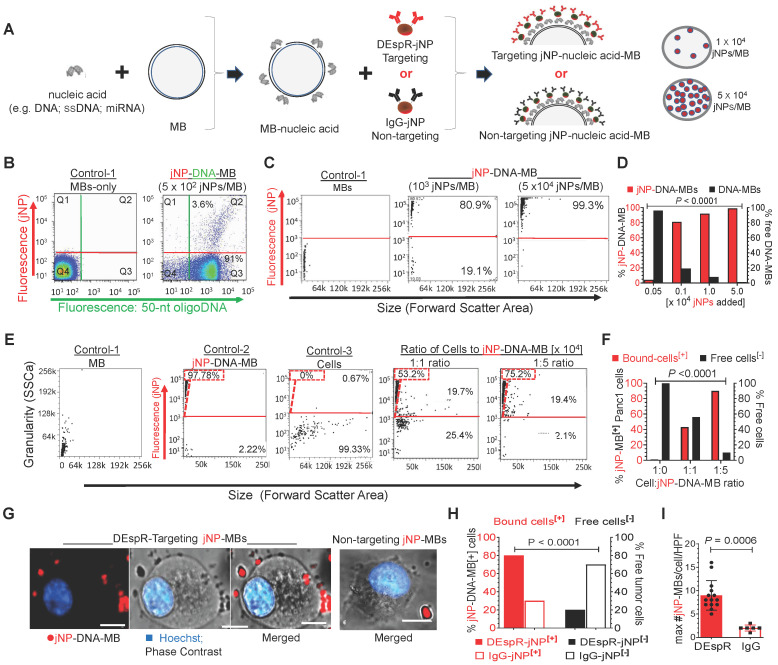
***In vitro* analysis of Janus nanoparticle (jNP) targeting and carrier functions. (A)** Schematic diagram of stepwise self-assembly and directional orientation of jNPs on 1 µm diameter microbubbles (MB) with zeta potential average -3.2 ± 0.4, forming jNP-DNA-MBs with tunable amount of jNPs added per MB (e.g., 1 x 10^4^ or 5 x 10^4^ jNPs/MB). DEspR-jNP, targeting jNP via anti-DEspR antibody face; IgG-jNP, control non-targeting with isotype IgG targeting face. **(B)** Representative flow cytometry analysis of double-fluorescent jNP-DNA-MBs distinguished from single-fluorescent and non-fluorescent MBs, using 5 x 10^2^ jNPs/MB. Y-axis: red-fluorescence intensity; X-axis, green-fluorescence; Control-1: non-fluorescent microbubbles (MBs) in quadrant 4 (Q4), red-fluorophore labeled jNPs (jNP), green-fluorophore labeled single strand 50-nt oligoDNA (DNA); 4-quadrants with differential fluorescence attained by MBs: ± bound DNA, ± bound jNPs: Q1-Q4. **(C)** Representative flow cytometry analysis of jNP-DNA-MB assembly: Y-axis, fluorescence intensity of jNPs with fluorescent antibody layer; X-axis, forward scatter representing size. Left-panel: non-fluorescent microbubbles (MB); Middle-panel: fluorescent jNP-DNA-MBs with 10^3^ jNPs/MB; Right panel: fluorescent jNP-DNA-MBs with 5 x 10^4^ jNPs/MB. Fluorescence intensity > 10^3^ above red horizontal line. **(D)** Contingency group analysis graph of jNP concentration-dependent self-assembly of jNP-DNA-MBs: fluorescent self-assembled jNP-DNA-MBs (solid red bars), non-fluorescent, non-assembled or free DNA-MBs (solid black bars); contingency chi square analysis, *P* < 0.0001. **(E)** Flow cytometry analysis of DEspR-targeting jNP-DNA-MBs binding to pancreatic tumor (Panc1) cells using different cell-to-[jNP-DNA-MB] ratios, X-axis: size indicator forward scatter area. Control-1, non-fluorescent MB only, Y-axis: side scatter granularity. Control-2, red-fluorescently labeled jNP-DNA-MBs only; Control-3: panc1 tumor cells only; cell-complex formation with 1:1 ratio of Panc1 cells to DEspR-targeting jNP-DNA-MBs; and with 1:5 cell-complex ratio using 1 x 10^4^ DEspR-targeting jNP-DNA-MBs (panels with Y-axis: fluorescence intensity of jNPs from labeled antibody layer). Free jNPs gated (dashed red triangle with corresponding % in dashed rectangle); free cells below the red horizontal line; jNP-DNA-MB bound cells: fluorescent = above red line. **(F)** Contingency group analysis graph of flow cytometry results comparing % bound vs % free cells exposed to jNP-DNA-MBs at 0, 1:1, and 1:5 ratio of cells-to-jNP-DNA-MBs: % bound cells (solid red bars jNP-MB^[+]^), and % free cells (solid black bars). Chi square analysis, n = 5000 cells, *P* < 0.0001. **(G)** Representative fluorescence microscopy images of a Panc1 tumor cell with multiple bound jNP-DNA-MBs (~ 1 µm diameter MBs). Red: fluorescently-labeled jNP-DNA-MBs, blue: Hoechst nuclear stain, bar = 5 µm. **(H)** Contingency group analysis graph of % bound cells^[+]^ with bound DEspR-targeting jNP-DNA-MBs (solid red bar DEspR-jNP^[+]^), or with non-specific bound non-targeting isotype (IgG) jNP-DNA-MBs (open red bar: IgG-jNP^[+]^); compared with free cells (solid black bar: DEspR-jNP^[-]^ cells); open black bar: IgG-jNP^[-]^ cells); contingency chi-square analysis: *P* < 0.0001; n = 80 cells exposed to DEspR jNP-DNA-MBs); n= 30 cells exposed to IgG jNP-DNA-MBs. **(I)** Comparison of cell-targeting showing maximum number (max #) of fluorescently labeled jNP-DNA-MBs bound to Panc1 tumor cells comparing DEspR-targeting jNP-DNA-MBs (solid red bar) *vs* isotype IgG non-targeting jNP-DNA-MBs. Mann Whitney test: *P* = 0.0006; DEspR-targeting (solid red bar) n = 14 cells; non-targeting IgG-isotype (open red bar) n = 6 cells (cells with no jNP-DNA-MBs excluded here).

Next, we verified jNP-DNA-MB serial-assembly (**Figure 3A**). Flow cytometry (FCM or FACS) analysis validates stepwise assembly: unlabeled MBs, single-fluorescent DNA^AF488^-bound MBs, and double-fluorescent jNP^AF568^-DNA^AF488^-MBs. Notably, flow cytometry shows DNA^AF488^ bind to majority of non-fluorescent MBs shown in quadrant-3, and 10^2^ jNPs^AF568^/MB bind completely to DNA^AF488^-MBs, forming dual-fluorescent jNP^AF568^-DNA^AF468^-MBs in quadrant-2 (**Figure 3B**).

To obtain a higher yield of jNP-DNA-MBs and test for concentration-response specificity, we added increasing amounts of jNPs to MBs (**Figure 3C**) and analyzed for self-assembled jNP-DNA-MBs by flow cytometry. In contrast to non-fluorescent MBs (**Figure 3C-left panel**), increasing the jNP-to-MB ratio to 10^3^, 10^4^, and 5 x 10^4^ jNPs/MB increases levels of fluorescent jNP-DNA-MBs: 80%, 97%, and 99%, respectively (**Figure 3C-D**). Concordantly, flow cytometry side scatter analysis of granularity shows increasing granularity of jNP-bound MBs in a concentration-response manner consistent with increasing jNP-binding to MBs (**Figure S2-A**). Flow cytometry with 1 and 15 μm 'bead marker' standards confirms similar size range of MBs per vial and verifies non-autofluorescence of MBs and relatively constant 1-1.5 μm diameter of MBs up to 6 h from reconstitution (**Figure S2B**).

### jNP-DNA-MB* in vitro* multifunctionality

To test jNP cell-targeting functionality, we prepared antibody targeting-jNPs that will bind the dual endothelin1/VEGFsp receptor (DEspR) expressed on PDAC tumor vascular endothelial and tumor cells 37. *In vitro*, Panc1-cells gain fluorescence upon binding of anti-DEspR^AF594^-jNP-DNA-MBs in a concentration-dependent manner, in contrast to non-fluorescent MBs and cells alone (**Figure 3E**). Notably, 1:1 cell-to-[jNP-DNA-MB] ratio bind 40% of total Panc1 cells, whereas 1:5 ratio bind ~ 90% of total cells (**Figure 3E-F**). Confocal microscopy confirms binding of DEspR^AF594^-targeting jNP-DNA-MBs to > 80% of Panc1-cells, with most cells exhibiting multiple jNP^AF594^-DNA-MBs (**Figure 3G-H**). In contrast, non-targeting isotype IgG^AF568^-jNP-DNA-MBs bind significantly fewer than 30% of Panc1 cells and with significantly fewer bound IgG^AF568^-jNP-DNA-MBs per cell (**Figure 3G-I**).

**Figure 4:**
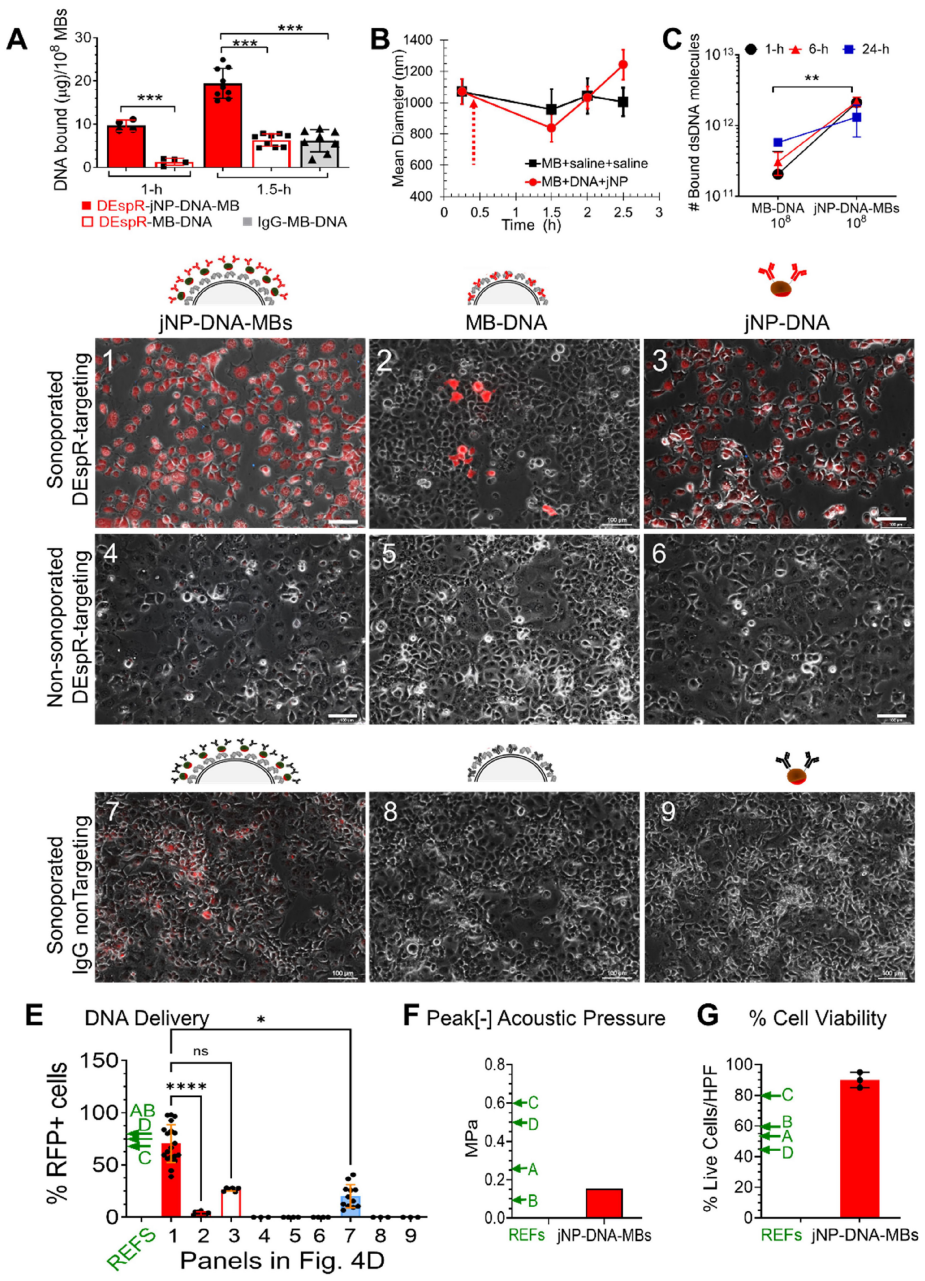
** Analysis of jNP-MB payload-binding, stability, and delivery functions. (A**) Payload capacity measured as bound DNA (mg DNA/10^8^ MBs) of self-assembled complexes at different durations (1 and 1.5 h) of incubation for self-assembly, data presented mean ± s.d. Solid red bar: DEspR-targeting jNP-DNA-MBs (10^8^ MBs per replicate: n = 4 replicates from 1 MB batch at 1 h, n = 9 replicates, 3 MB batches at 1.5 h); open bar: control DEspR-targeting (biotin-avidin) “Target-ready" MB-DNA (n = 4 replicates from 1 Target-ready MB batch at 1 h, n = 9 replicates from 3 Target-ready MB batches at 1.5 h); Solid grey bar, control non-targeting IgG-MB-DNA (n = 8). ***, P < 0.0001, 1-way ANOVA with Tukey's multiple pairwise comparisons. (**B**) Size-stability over time of self-assembled jNP-DNA-MBs compared with MBs. Hydrodynamic diameters (nm) measured by dynamic light scattering. Solid red circles, jNP-DNA-MB (n = 3 replicates, each with 10^8^ MBs); solid black squares, control MB-DNA (n = 3 replicates, each with 10^8^ MBs). Dashed arrow marks time of addition of jNPs after reconstitution of lyophilized MBs per manufacturer's specifications. (**C**) DNA-binding stability of jNP-DNA-MBs (n = 2 aliquots from 4 self-assembled mixtures comprised of 10^8^ MBs, 10^12^ jNPs, 30 μg DNA), *vs* control MB-DNA (n = 2 aliquots from 3 self-assembled mixtures, each with 10^8^ MBs, 30 μg DNA) x 3 time points: 1, 6, and 24 h measured as bound double strand DNA molecules (dsDNA). Two-way ANOVA (jNP-DNA-MB x time): row factor: MB-DNA vs jNP-DNA-MB ** *p* = 0.0002; column factor: time n.s. (**D**) Representative microscopy images with identical photo-exposures comparing *in vitro* fluorescence resulting from successful transfection of intact payload: reporter minigene-DNA construct for red fluorescent protein (RFP) to Panc1 cells 48 h after exposure. Three constructs represented in diagrams: jNP-DNA-MBs, control MB-DNA and control jNP-DNA are tested in 3 conditions: row-1: sonoporation of DEspR-targeting constructs, row-2: non-sonoporated but DEspR-targeting; row-3: sonoporated but non-DEspR targeting (non-specific IgG instead of anti-DEspR antibody). Panels 1-9 represent Panc1 cells subjected to 3 x 3 permutations: 3 constructs x 3 conditions. Cells exhibiting RFP-positive expression fluoresce red. Bar = 100 µm; identical experimental conditions: ~ 1:5 cell:MB ratio, DNA-MB ratio (30 mg DNA/10^8^ MBs); 10^4^ jNPs/MB used for DEspR-targeting and isotype IgG-non-targeting jNP-DNA-MB complexes. (**E**) Bar graph of % RFP-positive cells in peak RFP+ high power fields with > 50 cells/field (n = 3-19 fields) from three independent experiments (4 sonoporation sites, 1 control site per experiment) of study groups represented in panels 1-9. Kruskall Wallis non-parametric ANOVA *P* < 0.0001; panel 1: n=19 fields; panel 2: n=12 fields; panels 3, 4, 6: n=3 fields, panel 5: n=4 fields; panel 7: n=12 fields; panels 8, 9: n=3 fields. Post-hoc test: Dunn's multiple comparisons test, *, *p* < 0.05; ****, *p* < 0.0001. (**F**) Peak negative acoustic pressure (red bar) used in sonoporation of jNP-MB complexes compared with reported acoustic pressure levels in studies of CMBs by others (green ←) in References (REFs) A-D. (**G**) Bar graph of % cell viability before (100%) and after sonoporation of Panc1 tumor cells transfected using DEspR-targeting jNP-DNA-MBs delivering RFP-minigene DNA (n = 3 independent experiments). REFs, corresponding reference levels from published comparator CMBs: A, B, C, D, notated with green arrows in panels 4E, 4F, 4G are references 38-41, respectively.

**Figure 5:**
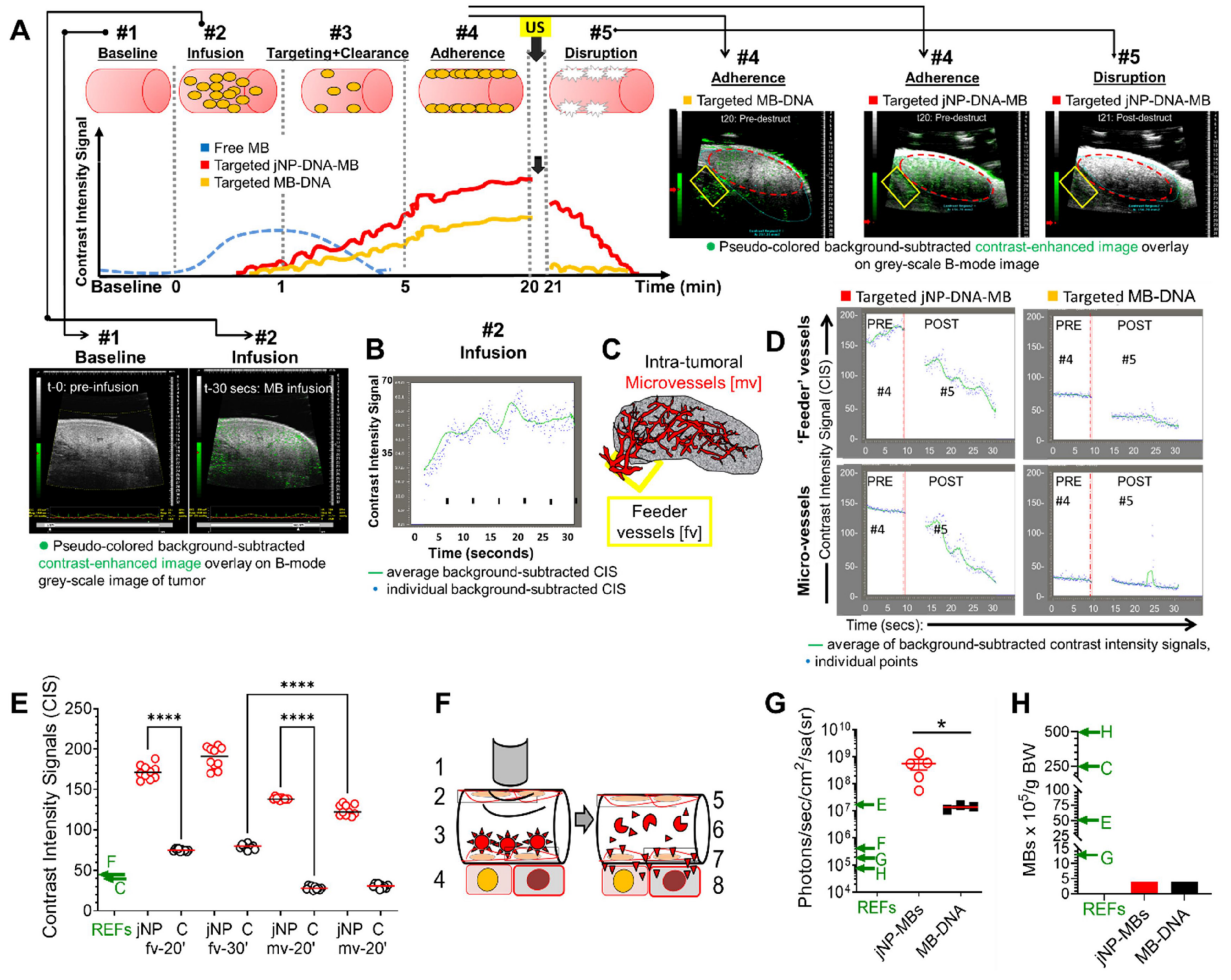
***In vivo* analysis of jNP-MB theranostic functionality: contrast-enhanced ultrasound molecular-imaging and delivery of reporter-RFP minigene. (A)** Diagram of molecular imaging sequence, with key events marked #1-#5 in series. Arrows connect to corresponding representative contrast-enhanced ultrasound images: overlay of B-mode (grey-scale image) and contrast-enhanced images (pseudo-colored green image) of spontaneous rat mammary tumors showing baseline (#1), during bolus infusion (#2) comparing control DEspR-targeted MB-DNA (Targeted MB-DNA, yellow line) and DEspR-targeted jNP-DNA-MBs (red line) during adherence (#4) and after disruption (#5) of MBs. Free MBs, dotted blue line are typically cleared by 5 min. **(B)** Corresponding time intensity curve generated during infusion (#2), average (green line), individual signals (blue dots). **(C)** Diagram depicting regions of interest (ROI) for time-intensity analysis of molecular imaging done on mammary tumors: ROI of intratumoral microvessels (mv, red vessels, dashed red oval in A#4, A#5) and of tumor feeder vessels at base of tumor (boxed yellow here and in A). **(D)** Representative time-intensity curves of background-subtracted contrast intensity signals (CIS) in designated tumor-ROIs at pre-destruct (pre) and post-destruct (post) comparing DEspR-targeting jNP-DNA-MBs (Targeted jNP-DNA-MBs) and control DEspR-targeting (biotin-avidin) MBs (Targeted MB-DNA) in extra-tumoral feeder vessels (fv) and intratumoral microvessels (mv). Timepoint of high-power ultrasound MB-destruct sequence (dashed line) demarcating pre- and post-destruct CIS. Green line, average of background-subtracted contrast intensity signals (CIS, blue dots) representing contrast-enhanced signals from adherent targeted-MBs in pre-destruct phase, and confirmation of adherent MBs after MB-destruction in post-destruct phase, determined via VisualSonics Contrast software. **(E)** Quantitative analysis of average CIS in the 10 s pre-destruct sequence, comparing DEspR-targeting jNP-DNA-MBs (jNP, open red circles) *vs* control (C) non-jNP DEspR-targeting MB-DNA microbubbles (C, open black circles) in two ROIs: extratumoral feeder vessels (fv) and intratumoral microvessels (mv). At t-20 (and t-30 min), CIS-levels represent mostly if not only DEspR-bound adherent MBs, as shown at the end of post-destruct level. One-way ANOVA *P* < 0.0001; ****, Tukey's multiple pairwise comparison *P* < 0.0001, 8 groups, n = 10 average CIS-levels/group representing 3-4 per second averages during pre-destruct phase, from 3 independent experiments using spontaneous mammary tumor rat model. Average CIS values taken from both tumor-ROIs: extra-tumoral feeder vessels (fv) and intra-tumoral microvessels (vs) at two imaging sessions (t20- and t30 min). **(F)** Diagram of key events in sonoporation of targeting jNP-DNA-MBs in intratumoral microvessels: pre-sonoporation #1-#4: #1, sonoporator; #2, endothelial cells in microvessel; 3, adherent jNP-DNA-MBs after clearance of unbound MBs; 4: tumor cells in cancer-microvascular niche; ➔, after sonoporation #5-#8: #5, non-injured endothelial cells; #6, disassembled jNPs and MBs and disrupted insonated MBs; #7, jNP-DNA released from MBs and direct entry into cytosol through transient “sonopores” that seal subsequently in conditions with no acoustic injury; #8, heterogeneous tumor cells in perivascular cancer niche transfected with jNP-DNA functional RFP-minigene (red inverted triangles). **(G)** Graph of IVIS-generated peak reporter-function fluorescence *in vivo* comparing DEspR-targeting jNP-DNA-MBs (open red circles, n = 5 tumors, min 4.7 x 10^7^ to 1.4 x 10^9^ photons/s/area) and control DEspR-targeting MB-DNA (solid black squares, n = 3 tumors); *, p = 0.036 two-tailed Mann Whitney test. Peak fluorescence units from published reports of *in vivo* delivery using CMBs are noted as relative reference points (REFs) with arrows: E, F, G, H 45-48, respectively. Reference F is ICAM-1 targeted; E, G, H utilize default liver-uptake. **(H)** Comparison of number of MBs used per gram body weight (#MBs: 4 x 10^5^/g BW for jNP-MBs and control MB-DNA) used for *in vivo* delivery of reporter function genes comparing jNP-MBs used in 200-250 g rat models, with published CMBs (REFs C, E, G, H are 40,45,47,48, respectively, used in 20-25 g mouse models.

**Figure 6:**
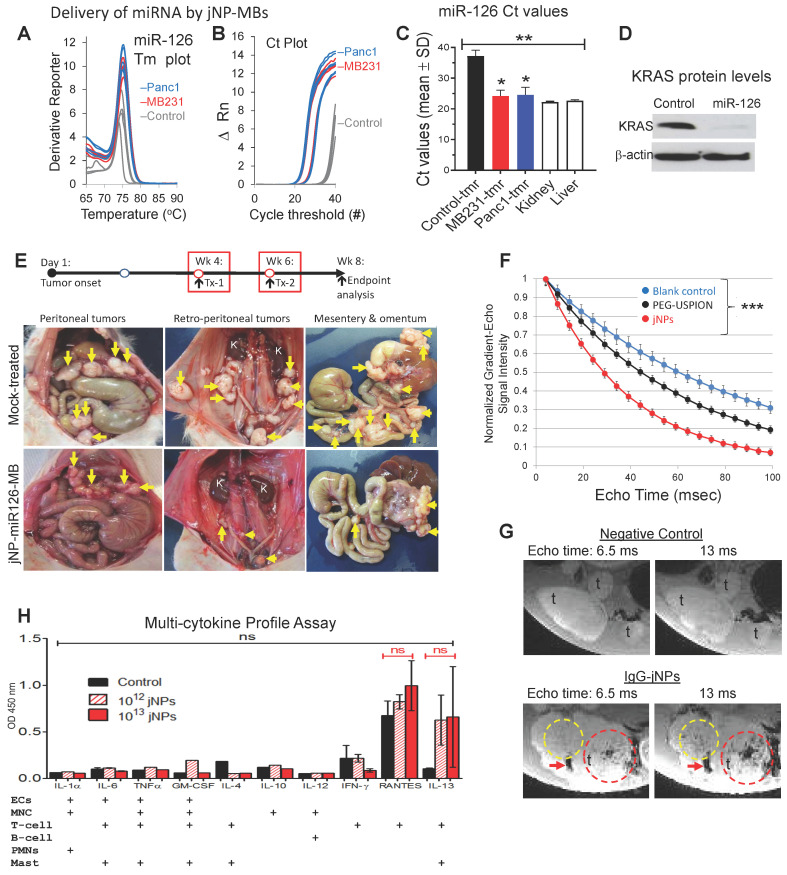
*In vivo* analysis of jNP-mediated targeted delivery of miRNA and contrast-enhanced MR-imaging. (**A**) Plot of melting temperatures (Tm) derived from real-time qRT-PCR analyses of miRNA-126 corroborating miRNA-126-specific amplification to detect miRNA-126 in rat xenograft tumors 48-h after sonoporation with DEspR-targeted jNP[miRNA-126]MBs: using 10^8^ MBs, 10^12^ jNPs, 27 µg of ds[miRNA-126]-mimic compared to negative control tumors (non-sonoporated, non-infused). T_m_ plots from different samples are identical and consistent with expected Tm for miRNA-126 ~ 75.4°C. (**B**) Real-time qRT-PCR cycle threshold (Ct) plots of miRNA-126 comparing sonoporated breast (red: MB-231-CSC) and pancreatic (blue: Panc1-CSC) xenograft tumors and negative control (grey: non-sonoporated, non-infused) tumors; low Ct indicate high miRNA-126 levels. (**C**) Bar graph of Ct values, means ± sd; **, *P* < 0.001 one-way ANOVA followed by Holms Sidak multiple pairwise comparison of control tumor (tmr) *vs* tumor tissues individually, *, *P* < 0.05. Control tumor (non-treated, solid black bar), control normal kidney and liver (n = 4/group) (open bars); sonoporated for miRNA-126 delivery: MB 231 TNBC-mammary xenograft tumor (n = 4) (solid red bar), Panc1 pancreatic cancer xenograft subcutaneous tumor (n = 6) (solid blue bar). (**D**) Representative Western blot analysis of miRNA-126's target KRAS protein shows decreased KRAS level 48 h after delivery of miRNA-126 by sonoporation; b-actin protein levels serve as internal control. (**E**) jNP-MB miR-126 *in vivo* testing in a xenograft tumor model of pancreatic peritoneal metastasis. Diagram of experimental timeline of tumor establishment and miR-126 delivery. Representative necropsy pictures at study endpoint comparing control mock-treated and jNP-MB miR-126 treated rat with xenograft Panc1-CSC derived peritoneal metastasis. Yellow arrows point to tumors. (**F**) Magnetic resonance studies of gradient-echo signal intensity versus echo time (TE), from 10-100 milliseconds (ms), for IgG-jNPs (solid red circles) and precursor PEG-uspion phantoms (solid black circles), both at 5 x 10^10^/mL in 1% agar, and control blank 1% agar phantom (solid blue circles). Data points show mean ± s.d. over 60 pixels in each phantom. Each curve was normalized so that peak signal at TE = 4 ms is equal to 1. jNPs exhibited shorter T2* values (mean ± sd: 35.2 ± 1.3 ms) compared with precursor PEG-USPIONs (57.6 ± 2.9 ms) and control blanks (82.2 ± 4.6 ms), *** *P* < 0.001, two-way ANOVA (subtype x time) repeated measures. (**G**) Representative *ex vivo* magnetic resonance (MR)-images (MRI) of pancreatic peritoneal tumors obtained using identical MRI settings and digital image settings 24-h after infusion of jNPs compared with control no jNPs infused. Regions containing high concentrations of jNPs showed hypointense (dark) signals at TE=6.5 ms, and this effect was amplified at TE=13 ms (compared area in dashed yellow and red circles, and area with red arrow). The control samples (no jNPs) did not show similar signal dropouts indicating presence of USPIONs in jNPs. In all images, t = tumor. (**H**) ELISA levels of key cytokines/chemokines (IL-1a, -6, -4, -10, -12, 13: interleukins; TNFa: tumor necrosis factor alpha, GM-CSF: granulocyte macrophage colony stimulating factor, IFN-g: interferon gamma, RANTES: Regulated on Activation, Normal T Cell Expressed and Secreted (or CCL5) produced by cells which are exposed to jNPs in the circulation such as ECs, endothelial cells, MNC, monocytes, T- and B-cell leukocytes, PMNs, neutrophils; Mast, complement-activating mast cells. Statistics performed: two-way (jNP-dose x cytokine levels across different cytokines) ANOVA (ns, not significant); n = 2 rats/group x 3 groups: 10^12^ jNP infusion (red hashed bars), 10^13^ jNPs infusion (solid red bars), and no jNP-infusion (solid black bars) negative control rats. RANTES and IL-13 show elevation but not significantly different between groups likely due to rat-specific wide-variations.

Having shown self-assembly (**Figure 3**), we next tested putative advantages of jNP-DNA-MB in DNA-delivery via ultrasound-mediated sonoporation 8 *in vitro*. First, DEspR-targeting jNP-DNA-MBs exhibit a greater protected-payload capacity over DEspR-targeting MB-DNA and MB-DNA controls (**Figure 4A**). Commercial MBs maintain equivalent MB-size across batches (**Figure 4B**) and stability of DNA-binding (**Figure 4C**) over time. Second, Panc1-cells sonoporated with anti-DEspR-jNP-DNA-MBs exhibit significantly more fluorescent cells (**Figure 4D-1**) than MB-DNA (**Figure 4D-2**) and jNP-DNA controls (**Figure 4D-3**), P < 0.0001 (**Figure 4E**). No fluorescence signal is detected in non-sonoporated cells (**Figure 4D-4-6**). Third, increased treatment efficacy is observed in targeted JUMPs (**Figure 4D-1-3**) over non-targeted controls (**Figure 4D-7-9**). Moreover, compared with cationic microbubbles (CMBs) reported by others 38-41, jNP-DNA-MBs exhibit higher % cell-transfection efficiency (**Figure 4E**), maintain a low peak negative acoustic pressure for sonoporation (**Figure 4F**), and exhibit higher % cell viability (**Figure 4G**).

### *In vivo* theranostic molecular imaging and delivery of DNA

Using an immunocompetent rat spontaneous mammary tumor model to better simulate tumor vascularization, we studied theranostic ultrasound molecular (DEspR-targeted) imaging and delivery of DNA-payload to mammary tumors (**Figure 5**). We used an experimental timeline that waits for clearance of unbound MBs at around t-5 min 42,43 to ascertain ultrasound-mediated imaging and sonoporation only of endothelial-adherent DEspR-bound MBs - whether DEspR-targeting jNP-DNA-MBs or DEspR-targeting MB-DNA (**Figure 5A**). After documenting infusion of MBs (**Figure 5A-diagram and image panels #1, #2, and Figure 5B**), we assessed ultrasound contrast-enhanced imaging of DEspR+ tumor feeder and intratumoral microvessels 20 and 30 min from infusion (t-20, t-30) (**Figure 5A-#4 diagram and image, and Figure 5C diagram**).

Ultrasound contrast-enhanced imaging of DEspR-targeted tumor blood vessels with anti-DEspR-jNP-DNA-MBs exhibit significantly greater background-subtracted CIS-levels at t-20 and t-30 min compared to control anti-DEspR-targeting MB-DNA (**Figure 5D-E**). While jNP-DNA-MBs exhibit a slower decline in CIS-levels after ultrasound destruction, CIS levels still reach baseline levels equivalent to control DEspR-targeting MBs after 15 s (**Figure 5D**). Comparative analyses of the average CIS-levels detected in feeder and intratumoral microvessels at t-20 and t-30 min after infusion show significant differences between jNP-DNA-MBs and MB-DNA controls (**Figure 5E**). Additionally, jNP-DNA-MBs attain higher average CIS-levels than CIS-levels reported in published tumor-targeting CMB studies (**Figure 5E**). Concordantly, the average CIS levels detected in feeder *vs* intratumoral microvessels are significantly higher for both jNP-DNA-MB and control (C) MB-DNA systems at both t-20 and t-30 min respectively (**Figure 5E**), consistent with impeded MB-oscillation in smaller microvessels 44. No empirical adverse effects were observed.

Having documented DEspR-targeting by ultrasound molecular imaging, we next compared delivery of red-fluorescent protein (RFP) DNA-construct by jNP-DNA-MBs compared with MB-DNA controls using identical DEspR-targeting. Forty-eight h after establishing baseline 'zero' fluorescence (**Figure S3**) and sonoporation of both DEspR-targeting MB-systems using identical protocols to deliver RFP-minigene to spontaneous rat mammary tumors (**Figure 5F**), *in vivo* imaging system (IVIS) analysis detects tumor fluorescence distinct from baseline (**Figure S3**). RFP-expressed fluorescence levels are greater in mammary tumors sonoporated using DEspR-targeting jNP-DNA-MBs compared with DEspR-targeting MB-DNA controls (**Figure 5G**), and compared with published CMBs 40, 45-48 (**Figure 5G**), while using a smaller number of MBs per gram body weight (**Figure 5H**).

### Modular targeted delivery of miRNA by sonoporation

To demonstrate modular payload-delivery in two xenograft tumor models - breast cancer KRAS^G13D^-mutant MB-MDA-231 cancer stem-like cells (CSCs) and pancreatic cancer KRAS^G12D^-mutant Panc1 CSCs - we substituted miR-126 for RFP-DNA in JUMP self-assembly. The tumor-suppressor miRNA-126 (miR-126) is decreased in human breast and pancreatic cancers, resulting in increased pro-metastatic KRAS (Kirsten rat sarcoma viral oncogene homologue) levels 49-51. Restoration of tumor-suppressor miR-126 levels sufficient to effect decreases in downstream KRAS levels will only be achieved when delivering sufficient functionally active miR-126 to enable necessary interactions with RNA-induced silencing complex (RISC).

After ascertaining miRNA-126 amplification products by documentation of expected ~ 74.8 ºC melting temperature for miR-126 (**Figure 6A**), real-time qRT-PCR detected > 1000X increase in miRNA-126 levels (low Ct values) in xenograft MB-231 mammary and Panc1 pancreatic subcutaneous tumors sonoporated with DEspR-targeting jNP-miR-126-MBs (**Figure 6B**). In contrast, very low expression levels of miR-126 are observed in negative control tumors (significantly higher Ct values, *P* < 0.0001) (**Figure 6B-C**). Notably, delivery of miR-126 achieves restoration of levels to those observed in normal liver and kidney tissues (**Figure 6C**).

To determine functionality of delivered miR-126, we performed comparative Western blot analyses of KRAS and β-actin levels, which shows decreased KRAS-levels up to 2.8-fold in Panc1-CSC xenograft tumors (**Figure 6D, Figure S4**) but no changes in control β-actin levels (**Figure 6D, Figure S4**). To further test delivery functionality, we investigated whether anti-DEspR-jNP-miR-126-MBs can deliver sufficient miR-126 to pancreatic peritoneal metastatic tumors (PPmets) and alter the course of PPmet growth and dissemination. Using identical experimental conditions, we infused and sonoporated anti-DEspR-jNP-miR-126-MBs with the ultrasound probe - covering all 4 quadrants of the abdomen. At 1 week and 6 weeks after PPmet initiation, followed by endpoint analysis at week 8 from tumor onset (**Figure 6E**), there is a notable decrease in PPmet number, size and spread in the peritoneal and retroperitoneal space (**Figure 6E**).

### Advantage of the jNP-design in magnetic resonance imaging

To demonstrate multi-modal capabilities in our novel JUMP system, MR-analysis shows jNPs exhibit shorter T2*-relaxivity compared with precursor PEG-USPIONs and control blanks across a range of 10-100 milliseconds echo times, *P* < 0.001 (**Figure 6F**). To see if this would translate to MR-contrast enhanced imaging, we next tested whether non-targeting IgG-jNPs localize to tumors by enhanced permeability and retention (EPR)-effects, which would require a minimum of 6 h of high plasma concentration 52 and provide contrast-enhanced MR-imaging. *Ex vivo* (9.4 Tesla) MR-imaging shows control tumors without jNPs do not exhibit MR-T2*-hypointensity at short and extended echo times (**Figure 6G**). In contrast, PPmets from rats infused with 10^12^ jNPs/rat exhibit increasing intra-tumoral MR-T2*-hypointensities with increasing 6.5 to 13 ms echo times (**Figure 6G**) 24-h after jNP infusion.

Non-targeted tissues (e.g. normal gut tissues) do not exhibit MR-T2*-hypointensity signals (**Figure 6G**), similar to liver and kidney (**Figure S5**). MR-T2*-hypointensities in the liver at 24 h exhibit structural patterns consistent with clearance via the hepatic Mononuclear Phagocyte System, as expected (**Figure S5**). Likewise, detection of MR-T2*-hypointensities with anatomical structural features in the kidney cortex - but not in the renal medulla or pelvis (**Figure S5**) - suggest jNP presence in the microvascular space or endothelial glycocalyx, but non-clearance through the kidney, which requires NPs < 6 nm 53.

### jNP safety advantage

To gain insight into *in vivo* jNP safety profiles, we infused 10^12^ jNPs into two groups of rats and monitored daily up to 2 weeks: (1) ~ 6 month old hypertensive stroke-prone rats infused with 10^12^ jNPs testing jNP-safety in the presence of primed neutrophils and microvascular endothelial activation; (2) ~ 6 month old normal rats infused with 10^13^ jNPs. Neither group exhibit adverse events. Analysis of cytokines/chemokines released by different leukocytes (monocytes, neutrophils, T-cells, mast cells) or activated endothelial cells in these rats show major proinflammatory cytokines increased in a cytokine storm (TNF-alpha, IL-6), none of which are elevated compared to control rats (**Figure 6H**). Observed elevation of RANTES and IL-13 are not significantly different from controls due to a wide range, indicating potential rat-specific responses (**Figure 6H**).

## Discussion

The covalently layered jNP-design attains ~ 3-fold smaller size than reported Janus nanoparticles 54 and displays multiple advantages not attained in other nucleic-acid delivery systems. First, DNA/miRNA delivery is attained *in vitro* and *in vivo* safely* using 6-fold less nucleic-acid payloads* (~ 30 μg/rat) and *10-fold fewer microbubbles* at 10^8^ MBs/rat compared to reported mouse studies using 200 μg DNA/mouse and 10^9^ MBs/mouse 48, or 500 μg DNA/mouse and 10^8^ MBs/mouse 9. These lower concentrations yielding successful molecular imaging and delivery contribute to observed *in vivo* safety profiles. Second, jNPs stably bind and protect DNA-payloads without decreasing delivery upon sonoporation of the jNP-payload-MB system: while jNPs can displace EtBr from its intercalation into DNA, EtBr cannot displace jNPs bound to DNA. This stable payload binding does not, however, block sonoporation-mediated delivery *in vitro* and *in vivo*. Third, jNP-payload-MBs are relatively stable without impeding microbubble acoustic properties, contributing to effective delivery by sonoporation of DNA/miRNA payloads to subcutaneous and peritoneal metastatic tumors at clinically safe ultrasound energies (153 kPa). Fourth, we demonstrate versatility and modularity of our system by using commercially available zwitterionic microbubbles 45 shown to be safe and able to bind DNA without need for cationic microbubbles (CMBs). This eliminates risk from cationic charge-induced complement activation and non-specific binding or adverse effects arising from cationic charges electrostatically binding to negatively charged cell membranes 55. This is particularly important in applications where sonoporation is used: all components of the assembled system must individually be safe. Finally, our system does not induce complement activation. Our data is similar to observations of inertness for L-PEI_25k_ polyplexes in mice 56, but is in contrast to studies using larger (> 25 kDa) or branched lipopolyplexes 56 and other SPION MR-contrast agents for tumor imaging, which have been shown to trigger inflammatory responses 32.

Furthermore, when we compare our JUMP system to published *in vitro* reports 38-40 of CMBs, jNP-DNA-MBs perform equivalently - if not better - in terms of peak percent-cell transfection (as measured by RFP fluorescence) and cell viability while using equivalent amounts of DNA payload and MBs. Likewise, jNP-DNA-MBs also show improved target-specific plasmid DNA-delivery and ultrasound contrast-enhanced imaging compared with reported studies of commercially available Micromarker and target-ready Micromarker MBs 57.

The *in vivo* detection of RFP-fluorescence after RFP-minigene delivery, and decreased KRAS protein levels after restoration of miR-126 levels demonstrate effectiveness of targeted jNP-payload-MB sonoporation as an endosomal-independent 8, 58 DNA/miRNA targeted delivery system. Observed tumor fluorescence indicates delivered reporter RFP-DNA was transcribed and translated. Moreover, when comparing *in vivo* published reports 40, 45-48 of CMBs, we find higher levels of RFP-fluorescence in our system for similar amounts of DNA payload and MBs. Observed restoration of miR-126 levels decreased KRAS protein levels. The potential of jNP system to restore levels of tumor suppressor miR-126 associated with cancer aggressiveness 49 opens a door to advancing miRNA therapy by overcoming limitations of neutral and CMB-delivery systems 38-41,45-48, and other nano-delivery systems relying on EPR 59,60, endocytic-uptake, and endosomal escape 61. The observed successful *in vivo* targeting of JUMP jNP-DNA/miRNA-MBs to DEspR+ tumor microvascular endothelial cells likely reflects the stable binding and internalization of anti-DEspR antibodies to endothelial cells 62 and Panc1 tumor cells 63.

The observed shorter MR-T2*-relaxivity of jNPs over precursor PEG-USPIONs 33, resulting in contrast-enhanced tumor MR-imaging suggest that spatial segregation of polycationic payload-face from antibody targeting face in jNP-design is key. JUMP's efficient architecture requires less material for theranostic functions: 10^12^ jNPs/rat or 5 ng Fe/100 g rat (*i.e.* 0.05 μg Fe/kg dose) is 10^3^ to 10^4^-fold less than clinically approved human doses for Resovist (60 nm diameter, 90 μg Fe/kg) and Ferridex (120-180 nm diameter, 560 μg Fe/kg), respectively. Better contrast-enhanced MR-imaging at 24 h compared with 4 h indicate sufficient jNP circulation to avail of tumor-selective EPR effects, which has been noted to need a minimum 6 h 52.

Absence of acute adverse events and non-activation of inflammatory pathways provide proof-of-concept for a promising safety profile of jNPs and jNP-payload-MBs. Our results using L-PEI_25kDa_ for the payload-face in jNPs is consistent with studies reporting the absence of acute adverse events for PEI-[DNA/siRNA] nano-polyplexes 26,27. This contrasts with 30-fold larger linear-PEI_750kDa_ and branched-PEI_25kDa_, which account for reported PEI-based polyplex toxicities 64. Moreover, delivery by sonoporation is orthogonal to endosomal uptake, establishing stimuli-inducible targeting specificity and therefore mitigating cellular toxicity associated with intra-endosomal inflammatory pathways reported for PEI-DNA/siRNA nano-polyplexes 26. Additionally, the jNP targeting antibody-face simultaneously serves as a 'pre-formed' protein corona, typically comprised of immunoglobulin, albumin and/or apolipoproteins 3. Integration of this selected targeting-antibody protein corona attains targeting functionality and avoids NP dysfunction and neoepitope toxicities from randomly “acquired” protein coronas.

In summary, JUMP allows for directional self-assembly and modular versatility for payload and targeting moieties and protection for nucleic acid payloads. Effectiveness of JUMP is demonstrated by lower amounts of reagents required to achieve similar or higher levels of targeted gene delivery or imaging *in vivo*. Enhanced multifunctionalities and safety profiles demonstrate proof-of-concept of JUMP as a cancer theranostic platform.

## Materials and Methods

### Preparation of Janus nanoparticles (jNPs)

Ultrasmall superparamagnetic iron oxide nanoparticles were assembled through covalent coupling of sequential layers. Monodisperse citric acid- coated Fe_3_O_4_ USPIONs (~ 10 nm in diameter) were functionalized to have a 4:1 PEG_2K/3.4K-NH2_ mixed brush 65, 66. A modified “layer-by-layer” technique 67 was used to prepare asymmetrically functionalized jNPs - nanoparticles with cationic PEI on one face, and an opposing antibody-face. Either targeted or non-targeted jNPs were prepared by using different antibodies on the antibody-face: targeted jNPs used a monoclonal antibody (mAb) moiety (anti-DEspR mAb) 37, whereas non-targeted jNPs were prepared using isotype IgG2 or non-specific IgG antibodies (Pierce Thermo Scientific, Rockford, IL). Stepwise layering was documented by changes in zeta potential or gain of fluorescence. To prepare fluorescently labeled jNPs for self-assembly studies with microbubbles (MBs), we used antibodies labeled either with red-fluorescent AF594 (jNP^AF594^) or with AF568 (jNP^AF568^) for the antibody-face. After preparation of mixed-brush PEG-coated USPIONs, which can be prepared in advance, the covalently coupled layering method for jNP synthesis included overnight incubation for targeting antibody conjugation and was then released from the mica sheet by adding salt as described below. Washing steps were performed between multiple covalent layering steps to eliminate leachables and excess reagent - PEI, antibody. Importantly, we use a nanoparticle concentration to prevent aggregation of the resulting Janus nanoparticles. A magnet was placed underneath the reaction dish to stabilize the PEI-USPIONs on the mica sheet to enable addition of subsequent layers and washing in between layers without losing SPIONs.

### Synthesis of jNPs

Janus nanoparticles were prepared using the following materials: iron tri(acetylacetonate) (99.9%), citric acid (CA; 99.5+%), methanol (99.8%), acetone, benzyl ether (99%), N-hydroxysuccinimide ester (NHS ester; 98%), oleic acid (OA; 90%), oleyl amine (OAm; 70%), 1-ethyl-3-(3-dimethylamino-propyl)carbodiimide hydrochloride (EDC; 97%) (Sigma, St. Louis, MO); 1,2- dichlorobenzene (DCB; 99%), N,N2-dimethylformamide (DMF; 99.8%), diethyl ether (99.9%), hexane (99.9%) (Acros, Morris Plains, NJ); ethanol (ACS grade) (Pharmco, Lees Summit, MO); amine-terminated polyethylene glycol (mPEG-NH_2_) with molecular weight 2000 Da (Laysan Bio, Inc, Arab, AL); NH_2_-mPEG-NH_2_ with molecular weight 3400 Da (Creative PEGWorks, Winston Salem, NC); RFP minigene (pTurbo FP635-N, Evrogen, Moscow, Russia); Alexafluor 594 or 568 labeling kits (Invitrogen, Carlsbad, CA, USA); anti-ratDEspR monoclonal antibodies (ProMab, CA); IgG2 isotype (Santa Cruz Biotechnology, Santa. Cruz, CA); MicroMarker microbubbles (Visual Sonics-Fuji, Inc., Canada).

Iron oxide core NPs were first synthesized according to the method described by Sun *et al* 65. Briefly, NPs were produced by an organic phase process reacting Fe(acac)_3_ and a long-chain alcohol, and then modified to yield monodisperse citric acid-coated Fe_3_O_4_ NPs about 10 nm in diameter. The NPs were then functionalized to contain a mixture of different polyethylene glycol (PEG) chains on their surface and attached as previously described 66. The mixed PEG brush was comprised of: molecular weight 2000 Da (PEG2000) and 3400 Da (PEG3400) in a 4:1 ratio. PEG2000 molecules were amine-terminated at one end, while PEG3400 molecules were amine-terminated at both ends. PEG chains were attached to carboxyl groups of citric acid on the USPION surface through N- hydroxysuccinimide (NHS) ester and 1-ethyl-3-(3-dimethylaminopropyl) carbodiimide hydrochloride (EDC) coupling 66 to form a mixed PEG brush coat around the USPION 28 with free amine groups only on PEG3400, referred to as the precursor USPION for production of Janus nanoparticles (jNPs).

The asymmetric functionalization method is based on previously reported layer-by-layer methods 68, 69. To prepare jNPs, a freshly 3x cleaved mica surface was placed in a 100 mm diameter petri dish. The square mica sheet was adjusted in size to fit in the dish. Magnets were attached to the underside of the petri dish to keep the magnetic USPIONs in place during the functionalization procedure. Upon placement in the petri dish, the negatively charged mica sheet was incubated with 0.1% solution of positively charged linear polyethyleneimine (PEI) of MW 25 kDa, washed and incubated with 5% glutaraldehyde solution to react with secondary amines on PEI. After a wash step, mixed-brush PEGylated USPIONs were added to the mica sheet with just enough USPIONs to cover the mica plate with a thin layer of USPIONs kept in place by the magnet during layer-by-layer functionalization and washes. The glutaraldehyde was allowed to react with amine groups on PEG3400, creating a monolayer of USPIONs bound on one side to the mica surface. After a wash step, the unbound face of the USPIONs oriented away from the mica was allowed to react with N-hydroxysuccinimide maleimide (NHS-MAL) at 100 mg/mL to convert free amines on the PEG3400 to a MAL.

After a wash step, targeting anti-DEspR antibodies or control non-targeting non-specific IgG were added to USPIONs for test jNPs and control jNPs, respectively, and allowed to attach overnight during gentle agitation to PEG3400-MAL groups to attain optimal tethered antibody-receptor interactions 70. Attachment of the antibodies to the USPION was achieved by coupling MAL on USPIONs to thiol groups on cysteine residues in the antibodies to attach (based on exposed USPION surface volume and antibody 12 nm diameter) approximately 9-13 antibodies to each USPION. Upon attachment of antibodies to the USPIONs, the mica sheet was washed and the resulting Janus nanoparticles, jNPs, were released using 0.2 M NaCl, disrupting electrostatic interactions between the mica sheet and PEI. Upon release, the solution was diluted with deionized H_2_O to yield a final NaCl concentration of 0.15 M.

### *In vitro* characterization of jNPs

Cryo-transmission electron microscopy (cryo-TEM) was performed to visualize jNPs using a CM12 Transmission Electron Microscope (Philips Electron Optics, Eindhoven, Netherlands). AFM was performed on jNPs and on DNA-jNPs. Determination of jNP diameters by DLS was performed per manufacturer's specifications. Study of jNP binding to DNA-payload was done via ethidium bromide (EtBr) dye exclusion assay as described 71, 72, with jNPs and DNA mixed prior to addition of EtBr. The affinity and stability of jNP binding to DNA was tested by displacement of DNA-intercalated EtBr in a dose-responsive manner. EtBr dye displacement assay was carried out with DNA and EtBr mixed first in a 4:1 ratio, followed by addition of jNPs. Dose-response was done with increasing number of jNPs using different DNA concentrations.

### Verification of layer-by-layer functionalized jNPs

To verify different steps of the covalent layering process, tests were conducted at different points of the procedure. The synthesis of PEGylated USPIONs had previously been verified in our laboratory through transmission electron microscopy, dynamic light scattering, zeta potential, elemental analysis, and iron quantification 73. To verify that the USPION surface is functionalized with a mixed layer of PEG2000 and amine-terminated PEG3400, analysis of increases in hydrodynamic radius from a 100% PEG 2000 layer to an 80%/20% PEG2000/PEG3400-NH_2_ layer were measured on a Brookhaven 90Plus (Brookhaven Instrument, Holtsville, NY) using dynamic light scattering (DLS). Zeta potential was used to confirm the modification steps. To verify the attachment of PEI, USPIONs were released before attachment of the antibody, and zeta potential was measured. To confirm attachment of antibodies, the antibodies were fluorescently labeled, and fluorescence was measured upon release from mica and subsequent wash of the jNPs. Antibodies were fluorescently labeled using the Alexa Fluor® Protein Labeling Kit from Invitrogen.

### Quantitation of jNPs

The number of jNPs produced by our method was quantified using the following materials: thioglycolic acid 98%, hydroxylamine hydrochloride, 1,10-phenathroline, sodium citrate, concentrated H_2_SO_4_, and ammonium iron(II) sulfate hexahydrate (Sigma Aldrich, MO, USA). jNPs were quantified using an adapted method from Nitin *et al* 74. The number of jNPs was determined by quantifying iron (Fe^3+^) concentration in Fe_3_O_4_ nanoparticles (NPs). Iron (Fe^3+^) in Fe_3_O_4_ was reduced to Fe^2+^ by adding 2 µL of thioglycolic acid to a 100 µL sample. After 2 h, 200 µL of 10% hydroxyl amine, 300 µL of 0.25% 1,10-phenathroline, 15 µL of 2.5% sodium citrate, and 1 mL deionized water were added, resulting in a compound [Fe(Phen)_3_]^2+^. The concentration of Fe^2+^ was calculated by measuring UV-VIS absorbance at 510 nm on a Molecular Devices Spectramax UV-Vis reader (Molecular Devices, Sunnyvale, CA), and the concentration was determined from a calibration curve, [Fe^2+^] = (Abs- 0.0058)/0.105 ppm. The calibration curve was made by dissolving various amounts of ammonium iron(II) sulfate hexahydrate in a 2% solution of H_2_SO_4_, obtaining the [Fe(Phen)_3_]^2+^ complex and measuring the absorbance at 510 nm to create a standard curve. The number of jNPs in the tested solution was determined by using the density of Fe_3_O_4_ and the volume of a 10 nm diameter sphere to estimate the number of jNPs equivalent to the ppm of iron.

### Cryo-electron microscopy of jNPs

A 3 µL aliquot of a suspension of jNPs in PBS at an approximate concentration of 10^12^ particles/mL was placed on a freshly glow-discharged 75 Quantifoil (Jena, Germany) 400 mesh copper grid overlaid with a 3 nm thick solid carbon layer. The sample grid was held by self-holding forceps vertically attached to a nitrogen gas-driven plunger in a humidified plexiglass freezing station. The grid was blotted with oven-dried filter paper and immediately plunged into supercooled ethane 76. Vitrified samples were stored under liquid nitrogen in a cryogenic storage dewar. At the time of imaging, vitrified samples were transferred under liquid nitrogen into a Gatan Single Tilt Cryoholder using a cryotransfer station (Gatan, Pleasanton, CA) and inserted into a CM12 Transmission Electron Microscope (Philips Electron Optics, Eindhoven, The Netherlands). Images were collected at 120KV, 75,000 X, and a defocus of 1 u with a Tietz 1K x 1K pixel CCD camera (Gauting, Germany) using EMMENU4 Program. A low dose kit minimizes irradiation damage to the sample.

### Atomic Force Microscopy (AFM) Imaging of jNPs

AFM imaging was performed 77 using an Agilent 5500 AFM operated in intermittent contact mode (Acoustic AC (AAC) mode) using a Mac II cantilever (Agilent Technologies) with a resonant frequency of approximately 75 kHz and a spring constant of 2.8 N/m. AFM images were recorded at a resolution of 512x512 pixels at a line rate of 1 line/s. Scan sizes ranged from 300 nm x 300 nm up to 1.5 μm x 1.5 μm.

### Ethidium Bromide (EtBr) Dye Exclusion and Displacement Assays

The binding of jNPs to DNA was tested by ethidium bromide (EtBr) dye exclusion assay 35 based on the principle that EtBr fluoresces only when it intercalates into DNA base pairs. Fluorescence therefore indicates free DNA base pairs that are not bound to jNPs. To demonstrate specificity, increasing amounts of jNPs (0, 0.2, 0.6, 1.8 x 10^10^ jNPs) were mixed with two different amounts of DNA (100 and 250 ng) and tested for EtBr fluorescence at two time points: 10 min and 1hr. Fluorescence reads were done at 260 nm excitation/590 nm emission, and background autofluorescence of non-intercalated EtBr was subtracted. Afterwards, samples were then run in 0.8% agarose gels and fluorescence from EtBr intercalated into DNA was visualized with UV 260 nm excitation and photographed.

To further test affinity of jNPs for DNA, we used an EtBr displacement assay 36, based on loss of fluorescence upon displacement of EtBr from DNA base pairs (bp) by jNPs competitively binding to DNA. Aliquots of DNA plasmid in a 96-well plate (100 or 250 ng plasmid GFP-DNA per well) in physiological salt solution (pH = 7, 150 mM NaCl) were incubated for 10 min with a molar ratio of 4:1 bp:EtBr. Increasing amounts of a solution containing 1.22 x 10^13^ np/mL in 150 mM NaCl were added to aliquots of DNA plasmid and brought to a final working concentration of 200 µl with additional physiological salt solution. Fluorescence of intercalated EtBr was measured at Ex./Em. of 480/590 nm using a Spectramax M5 plate reader (Molecular Devices, Sunnyvale, CA), and relative fluorescence values are reported as the ratio of fluorescence intensity of the sample containing jNP to samples containing EtBr/DNA plasmid alone (n=6).

### Measurement of the number of antibodies on jNPs

Because the only protein moiety on jNPs is the antibody layer, direct measurement of protein by standard BCA assays will detect the number of antibodies per jNP using the known mass of an antibody: 150 kDa, or 2.5 x 10^13^ µg/antibody. Three independent batches of jNPs were analyzed.

Standard curve for protein measurements by BCA assay affirms that using a 1:20 dilution of three independent jNP batches allows measurement of protein per jNP within the ideal sensitive range of 1-40 μg/mL for the BCA assay. The BCA assay (MicroBCA™ Protein Asay Kit, Thermo Scientific) was done per manufacturer's specifications.

### Analysis of jNP stability in serum

Stability against aggregation and loss of particle functionalization was tested by observing the effective diameter of jNPs in 85% rat serum solution using Dynamic Light Scattering (DLS) and Atomic Force Microscopy (AFM). Briefly, 250 µL of jNP in 150 mM NaCl was mixed with pure rat serum for a final jNP concentration of 8.5 x 10^11^ particles/mL in 75% rat serum. Mean jNP diameters were determined via DLS using a Brookhaven 90Plus (Brookhaven Instrument, Holtsville, NY) over several time points: 0, 1, 3, and 24 h. Additionally, at each time point, 10 µl was removed from the sample and deposited on a mica slide under a magnet. After allowing jNPs to deposit on the surface for one hour, slides were rinsed with DI water and dried for 48 h before imaging using AFM, as described above. Due to the asymmetrical shape of the jNP, AFM effective diameter was reported as the longest diameter through the USPION core.

### AFM imaging of jNP with lambda phage DNA

To further document interaction of jNPs with DNA, we used AFM to directly image jNP binding to lambda phage DNA strands 78. 1 µL of a 1x10^13^ solution of jNP and 25 µl of 1 mg/mL phage DNA were co-adsorbed on a mica substrate under a magnet. After allowing 1 h for jNPs and DNA to interact and adsorb, the mica was rinsed with deionized water and air-dried for 48 h prior to AFM imaging. Images were taken as described above.

### Analysis of complement activation *in vitro*

This was performed in 80% human normal plasma spanning *in vivo* doses of jNPs measuring the Terminal Complement Complex, SC5b-9 per manufacturer's specifications (Quidel, Inc., VA) in triplicate, with 2 independent jNPs (n = 6/group) x 3 groups: 0/mL, 10^10^ jNPs/mL, and 10^13^ jNPs/mL.

#### *In vitro* measurement of Terminal Complement Complex, SC5b-9, in human plasma

Different versions of functionalization of the nanoparticles were tested for complement activation, these were: 1) the NPs with only the PEG brush, 2) NPs with PEG brush and PEI on one half, 3) jNPs with targeting antibody on one side and an opposite PEI side (the jNPs as used in experiments) and 4) an isotropic NP functionalized with non-targeting antibodies covering the surface of the NP. Measurement of the terminal complement complex (TCC, SC5b-9) was performed on jNPs in pooled, Na-EDTA anticoagulated, normal human plasma (Valley Biomedical Inc., VA) using the MicroVue SC5b-9 Plus Enzyme Immunoassay as per manufacturer's specifications (Quidel, Inc., CA). Compared to normal plasma, SC5b-9 levels were measured in 80% plasma containing 10^10^ jNPs/mL and 10^13^ jNPs/mL This range spanned the *in vivo* doses used per rat of jNPs for molecular imaging, MRI, and delivery (10^12^ jNPs/rat), and for studies determining levels of different cytokines and chemokines (10^12^ and 10^13^ jNPs/rat). We used the conversion equivalence of 4.5 mL plasma per 100 g rat. The absorbance of the samples was recorded at 450 nm, and the SC5b-9 complex concentrations were determined from a standard curve spanning 0.0 to 170 ng/mL. Measurements were done within the linear range of the standard curve. Normal pooled plasma with no jNPs served as reference for normal SC5b-9 levels and was found to be 428.1 ± 86 ng/mL. Kruskall-Wallis ANOVA and Dunn's multiple comparison test were performed to determine significance of differences, if any. Two different batches of jNPs, confirmed to have average 40-50 nm diameter, were analyzed in triplicate, n = 6.

### Assembly of jNP-MBs and properties of commercially available MicroMarker microbubbles

Stepwise self-assembly of jNP-MBs was done in physiological saline at room temperature with mild rotation by first incubating the nucleic acid payload with MBs and then adding jNPs to form jNP-MBs (specific nucleic acid payload is indicated in the name, e.g. jNP-DNA-MB). We used commercially available microbubbles, MicroMarker Contrast Agents (VisualSonics-Fuji, Inc). The specifications of the MicroMarker microbubbles reported in the literature are as follows 79-82: 1-3 μm in diameter; shell composition: phospholipids, PEG, fatty acids; N_2_C_4_F_10_ gas; amphoteric surface charge. MBs with streptavidin (Target-Ready MicroMarker Contrast Agents, Visual Sonics-Fuji, Inc) were used for control targeted MBs for *in vivo* molecular imaging and as control targeted DNA-MBs for *in vitro* DNA delivery studies. These were linked to biotinylated anti-DEspR mAb as targeting moiety. The Target-Ready MicroMarker Microbubbles (MBs) were selected as they have been optimized for rodents and characterized previously by different research groups 42, 43, 83-86: size range 1-3 μm in diameter and contain 7,600 streptavidin molecules/μm^2^. Target-Ready MBs have been validated for molecular imaging by different groups using antibodies targeting VEGFR2 and integrins, for delivery of DNA via sonoporation, acoustical behavior, and transfection efficiency *in vitro* 57, 87-89.

To generate jNP-DNA-MBs, MBs were first resuspended in normal saline (150 mM NaCl) for 10 min following manufacturer's specifications. Then the nucleic acid payload (*e.g.* 30 µg of RFP-plasmid DNA, 27 µg of miRNA-126, or 15 µg of single strand Alexa Fluor-488-labled oligoDNA) was incubated with 1 x 10^8^ MBs for 15 min, followed by incubation with jNPs for another 45 min before further use or analysis. The amount of jNPs added was varied from 5 x 10^2^, 10^3^, 10^4^, and 5 x 10^4^ jNPs/MB.

### FACS analysis of jNP-MB stepwise assembly

To document assembly of the jNP-MBs, we followed the identical stepwise assembly described above, and used fluorescent jNPs prepared using fluorescently labeled anti-DEspR monoclonal antibodies (Alexa Fluor AF594) as the targeting moiety in the covalent layering preparation of jNPs. Assembly of fluorescent jNPs with MBs-DNA would therefore result in fluorescent jNP-DNA-MBs as distinguished from non-fluorescent MBs-DNA on FACS analysis (LSRII flow cytometer, Becton Dickinson, Inc). Increased granularity was assessed by side scatter. Increasing coverage of the 2 µm diameter MBs by jNPs was tested by FACS analysis.

To directly demonstrate nucleic acid payload binding to microbubbles, we used commercially available 50 nucleotide-long AF488-labeled oligoDNA. After resuspension of commercially available, non-fluorescent microbubbles (MicroMarkers, VisualSonics-Fuji, Inc., Canada), an aliquot of 10^8^ MBs was mixed with 15 μg AF488-labeled oligoDNA for 15 min with gentle mixing on a rotator, and an aliquot was analyzed by FACS analysis to document gain of fluorescence of MBs upon addition of fluorescent DNA. Then fluorescent MBs-DNA were mixed with fluorescent jNPs (non-targeted AF568-labeled IgG (goat anti-rabbit IgG, Pierce Thermo-Scientific, Rockford IL)) for 45 min and then analyzed by FACS analysis (t-1 h; t-24 h) for dual fluorescent jNP-DNA-MBs to distinguish from single fluorescent DNA-MBs and unlabeled MBs.

### Measurement of DNA payloads

The DNA payload in DNA-MBs was measured as follows: After suspension of microbubbles (MBs, MicroMarker microbubbles, Visual Sonics Inc.), 10^8^ MBs were mixed with 30 μg dsDNA in 1 mL saline solution with gentle agitation until analysis timepoints. Analysis was done on 3 independent self-assembled samples at t-0 (baseline control), and after 1, 6, and 24 h.

For jNP-DNA-MBs: Using the same stock of MBs, 10^8^ MBs were mixed with 30 μg dsDNA, and 10^12^ jNPs in a final volume of 1 mL saline solution and incubated with gentle agitation until analysis at designated times: t-0 baseline, and at 1, 6, and 24 h. Analysis of bound DNA was performed at baseline, and at 1, 6 and 24 h. Analysis of bound DNA (plasmid DNA for red fluorescent protein minigene, 4707 bp) was performed by analyzing residual unbound DNA concentration in the saline buffer after MBs-DNA and jNP-DNA-MBs were allowed to float (30 min) and removed completely. DNA was measured by UV (Absorbance: 260 nm) spectrophotometry (NanoDrop ND-1000 UV-VIS Spectrophotometer) after phenol-chloroform and ethanol precipitations were performed on 2 aliquots (50 µL/aliquot) taken at designated analysis times (1, 6, and 24 h) from the different jNP-DNA-MBs (n = 4) and DNA-MBs (n = 3) self-assembled mixtures.

Bound DNA was determined by subtracting the % unbound DNA from the starting material. The number of DNA molecules was determined stoichiometrically: 1 µg 4707-bp long DNA has 9.1 x 10^11^ DNA molecules. Two-way [time vs ± jNP] ANOVA was performed to determine whether jNP-DNA-MBs have significantly more DNA payload than DNA-MBs given identical stocks and numbers of DNA and MBs.

### *In vitro* analysis of jNP-DNA-MB multifunctionality

Targeting and stable binding of targeted anti-DEspR-jNP-DNA-MBs to Panc1 cancer cells was documented *in vitro* by FACS analysis and epi-fluorescence microscopy of fluorescently labeled jNPs. Targeted jNP-DNA-MBs had anti-human DEspR monoclonal ab (mAb) (in-house development) as the targeting moiety for the jNP. DEspR is present on Panc1 tumor cells 37. Human pancreatic cancer (Panc1) tumor cells were cultured as specified (ATCC), tested for mycoplasma contamination as required for tissue culture and animal model experiments, and maintained at 37°C in a humidified 5% CO*_2_* incubator. *In vitro* delivery of red fluorescent protein (RFP) mini-gene DNA was done by sonoporation and gene expression assessed by epi-fluorescence microscopy *in vitro*.

#### Flow cytometry analysis

Panc1 tumor cells were isolated, washed, counted, and 10,000 cells were then incubated with fluorescent jNP-DNA-MBs at room temperature with gentle rotation at ~ 1:1 and at ~ 1:5 cell-to-jNP-DNA-MB ratio for 45-60 min. Targeting efficiency was analyzed and quantified by flow cytometry and FloJo analysis software, comparing the % of Panc1 tumor cells that gain fluorescence with those that did not. Detection of fluorescent cells would indicate successful targeting of cells by fluorescent jNP- DNA- MBs through anti-DEspR Ab interactions with the cognate receptor (DEspR) on Panc1 tumor cells 37.

#### *In vitro* analysis of jNP-DNA-MB binding to Panc1 cells under low flow

Panc1 cells were grown as described and harvested at 60% confluency using 5 mM EDTA in 1X PBS, washed, and then scraped with a sterile cell lifter. 5,000 cells were seeded onto each channel in an IBIDI 6-lane µ-slide VI 0.4 ibiTreat (ibidiGmbH, Germany) and allowed to attach and recover for 3 h in a 37°C CO_2_ incubator. Growth media was then replaced with saline containing 50,000 DEspR-targeted or non-targeted isotype jNP-DNA-MBs labeled with AlexaFluor 594 on the targeting antibody moiety. The jNP-DNA-MBs (targeted and non-targeted isotype) were allowed to interact with the cells for 30 min under ~ 1-2 mL/min flow (estimate ~ 2-4 dynes/cm^2^ shear in IBIDI micro-slide VI, ibidiGmbH, Germany), simulating tumor microvascular flow. Afterwards, the media was flushed with 1x phosphate buffered saline to wash away unbound fluorescent jNP-DNA-MBs. Cell nuclei were counterstained with Hoechst nuclear stain. Imaging was done using a Nikon epi-fluorescence microscope for red fluorescence (Alexa Fluor-labeled DEspR-targeted jNP-DNA-MB), blue fluorescence (Hoechst nuclear stain), and phase-contrast imaging to delineate cell membranes. Non-specific binding was analyzed using jNPs functionalized with Alexa Fluor 594-labeled IgG2 isotype antibody.

#### Analysis of jNP-MB payload delivery *in vitro*

Panc1 tumor cells were grown in an OptiCell closed cell-culture system (Nunc/Thermo Scientific, USA) with 75 µm thick gas-permeable membrane walls, which were demonstrated to allow ultrasound to pass through for: 1) imaging of ultrasound contrast-enhanced microbubbles (data not shown) using a Vevo770 ultrasound imaging system with contrast-enhanced capabilities (Visual Sonics-Fuji, Inc., Canada), or 2) for sonoporation using a 1 MHz SoniGene sonoporator (VisualSonics-Fuji).

Panc1 cells were seeded at ~ 700,000 cells for one side of an OptiCell cassette and allowed to grow for ~ 48 h to 50-60% confluency, or approximately 8 x 10^6^ cells. Following identical conditions, DEspR-targeted and isotype non-targeted jNP-DNA-MBs (30 µg DNA, 10^8^ MBs; 10^12^ jNPs) and DEspR-targeted and isotype non-targeted MBs-DNA controls (30 µg DNA/10^8^ MBs) were prepared as described above in 1 mL then added to 9 mL of serum-free DMEM, resulting in about ~ 1:10 cells-to-DNA-MB ratio. Targeted and non-targeted MBs-DNA were allowed to interact with cells for 30 min with constant vertical rotation of OptiCell systems to simulate *in vivo* shear/flow disturbance. Excess unbound targeted and non-targeted jNP-DNA-MBs, and targeted and non- targeted MBs-DNA, as well as any free jNPs, DNA and MBs were removed by one wash with media after 30 min, and new media was added.

Sonoporation was then performed on adherent jNP-DNA-MBs or MBs-DNA with the OptiCell cassette cell-side up/non-cell side down configuration using a 1 cm diameter SoniGene probe using the following settings: 1.5 watts/cm^2^ for 60 s, 50% duty cycle. The probe was applied from the bottom (*i.e.*, on the non-cell side-down of the OptiCell system) with ultrasound gel to establish contact. Sonoporation was performed on 8 independent 1 cm diameter sonoporation-sites in 2 different OptiCell culture systems per group, with a designated non-sonoporated area as a control in each OptiCell culture system for 2 designated non-sonoporated sites.

After 48 h, analyses of cell health and RFP-mediated fluorescence were done using a Nikon epi-fluorescent microscope as described above. We compared DEspR-targeted jNP-DNA-MBs to: 1) DEspR-targeted MBs-DNA, 2) non-targeted isotype jNP-DNA-MBs, and 3) non-targeted isotype MBs-DNA with or without sonoporation to determine differential efficacies in the same OptiCell culture system. Quantitative analysis was performed by determining the % fluorescent RFP-positive cells over total cells in 1-2 FOVs (0.54 mm^2^ area, with 130 to 500 total cells in the field) obtained using identical exposures in each of the 8 independent sonoporation sites, and in the 2 independent non-sonoporation sites per study group. Cell membrane-demarcated fluorescence was ascertained by photomicroscopy. Bar = 100 μm.

### *In vivo* analysis of jNP and jNP-DNA-MB multifunctionality

*In vivo* tests of jNP-DNA-MBs were done using identical component ratios validated *in vitro*. All animal experiments were performed according to Institutional Animal Care and Use Committee. Three rat tumor models were used: spontaneous mammary tumors in post-menopausal female F2-[Dahl S x Dahl R]-intercross rats for ultrasound contrast-enhanced molecular imaging and for DNA delivery; female nude rat (*Rnu/Rnu*) heterotopic, xenograft tumor models of human pancreatic cancer (Panc1, ATCC® CRL1469™) and breast cancer (MDA-MB-231, ATCC-HTB26) developed from Panc1- and MDA-MB-231 cancer stem- like cells (CSCs) isolated from each line as described 90. Tumor cells and CSCs were documented as mycoplasma-free and pathogen-free prior to *in vitro* and *in vivo* experiments. Sample size estimates of rat groups depended on the tumor model. For the post-menopausal spontaneous mammary tumors, reproducibility in tumors and availability of spontaneous tumors randomly assigned to test and controls determined the group size. For xenograft tumor models, sample size estimates were based on outcome measures' mean ± s.d. that will give *P* < 0.05 with power 0.8, alpha = 0.05. Rats were randomly assigned to study groups by alternating test-and-control assignments. This ascertained that test and control rats were contemporaneous.

#### Ultrasound targeted molecular imaging

Targeted molecular imaging was performed using identical conditions for test and control rats using a Vevo770 ultrasound contrast-imaging system for molecular imaging and the VisualSonics Contrast Mode software for analysis as validated by 42,86. Visual imaging of contrast was obtained with intensity scales optimized for visual overlay of contrast and 2D-B-mode images, but quantitation was performed on identical scales.

To coordinate tandem molecular imaging and sonoporation experiments using the same spontaneous mammary tumor rat, we obtained baseline ultrasound 2-dimensional B-mode imaging and baseline IVIS documentation of zero-fluorescence of tumors. Using anti-DEspR antibody-targeting of angiogenic endothelial cells previously validated by us 62, DEspR-targeting jNP-DNA-MBs and MB-DNA controls were each comprised of 30 μg DNA, 1 x 10^8^ MBs, and 1 x 10^12^ DEspR-targeted jNPs for jNP-DNA-MBs, or anti-DEspR antibody streptavidin-biotin coupling for DEspR-MB-DNA. After infusion and an intrinsic microbubble clearance period (about ~ 5-10 min) 42,43, target-bound microbubbles were visualized by contrast-enhanced imaging of tumor feeder vessels at the base of the tumor and intratumoral microvessels, using the Vevo770 contrast-enhanced imaging program and software analysis at different time points (t): t10-, t15-, t20-, and/or t30- min post-infusion.

We performed contrast-enhanced ultrasound imaging using identical settings for all tumors following precautions to minimize movement artifacts: rats were anesthetized, scanhead was immobilized using the Vevo770 scanhead-specific rail-system, and imaging was gated to respirations. We used the Vevo770 Contrast Mode parameters as per manufacturer's specifications similar to published description by Lee *et a*l. 84 and Willmann, *et al.* 43 except that we used a rat-appropriate scanhead - the RMV-16, and modified the timing of the contrast-imaging protocol with later timepoints. The RMV-16 scan head has a broadband frequency up to 23.5 MHz, axial resolution of 85 μm and lateral resolution of 215 μm at focal length 17.5 mm, field of view up to 33 mm. B-mode imaging was optimized with acoustic focus centered at the level of the spontaneous mammary tumor. Rats were maintained on 1.2-1.5% isoflurane during scanning; and all imaging settings were kept constant for all imaging sessions. Tail vein infusions of test and control MBs (total 10^8^ MBs/rat) were done with documentation of increased contrast intensity signals (CISs) from the inflow of MBs in the femoral artery. After 5 min, clearance of most if not all MBs from the circulation was validated in the femoral artery. At t-10, the first molecular imaging tumor scan was done gated to respirations and with the scanhead immobilized on the Vevo770 rail system to eliminate movement artifacts manifested as speckle variance. 100 ultrasonographic frames were obtained at a temporal resolution of 10 frames/s, followed by a destruction pulse (20 cycles, 10 MHz, mechanical index of 0.59) for 5 s followed by imaging for another 15 s (~ 150 frames) post-destruction. This was repeated at t-20, t-30 min - then followed by sonoporation shortly after (see below).

Image processing and quantification were done using the Vevo770 Contrast Mode analysis offline for regions of interest (ROI) corresponding to feeder vessels and intratumoral vessels. As described by Lyshchik *et al.* 2007 42, “image processing in the Vevo770 system relies on 2 sets of images: a pre-destruction set and a post-destruction data set”. Images from the pre-destruction set were paired to their partner images in the post-destruction set using an absolute sum-of-differences technique. Once the image pairs were calculated, the subtracted image was generated and displayed in shades of green on top of the B-mode image by a blending algorithm to provide a map for the spatial distribution of the ultrasound contrast agents retained by the tissues”. Offline analysis with the Vevo770 Contrast Enhanced Imaging software of operator-selected ROIs then plots contrast intensity signals (CIS) against time (time-intensity plots) demarcating pre-destruction and post-destruction CIS levels and indicating the average of CIS levels as a green line in the time intensity plots.

### *In vivo* analysis of DNA-payload delivery by sonoporation using DEspR-targeted jNP-DNA- MBs and MBs-DNA

Baseline IVIS images were obtained prior to ultrasound molecular imaging. After contrast-enhanced ultrasound molecular imaging, sonoporation was performed for both DEspR-targeted jNP-DNA-MBs and control DEspR-targeted MBs-DNA using the SoniGene sonoporator with a 1 cm- diameter 1 MHz probe with a half-value depth of 2.3 cm. Sonoporation was performed with the following settings: 50% duty cycle, for 1.5 watts/cm^2^, for 60 s, 153 kPa (Sonigene, Visual Sonics Inc, Canada) pulses until the whole surface of the tumor is sonoporated. Delivery and functional expression (transcription, translation) of the RFP-minigene was determined by imaging red fluorescence in tumors by IVIS (Xenogen, Inc) two days after sonoporation. Mock-sonoporated control (sonoporation, no microbubbles) was imaged concurrently with IVIS as a control. Quantitation was done using the IVIS quantitation software of photons/cm^2^/s (Xenogen, Inc).

### *In vivo* analysis of jNP-MB delivery of miRNA-126 to tumors *in vivo*

Assembly of miRNA payload onto jNP-miR-MBs and *in vivo* delivery was done identically as in the case for jNP-DNA-MBs. We used 27-30 µg miRNA/10^8^ MBs/10^12^ jNPs per rat (21-bp double-strand miRNA-126 mimic, Stem Loop ID: hsa-miRNA-126-5p; Ambion Life Technologies). Two-days after miRNA-126 delivery by sonoporation of targeted anti-DEspR-jNP-miRNA-126-MBs to nude rat heterotopic xenograft tumors, detection of full-length miRNA-126 was performed by miRNA-126-specific quantitative real-time RT-PCR analysis. KRAS protein levels were determined by Western blot analysis.

#### Micro-RNA Isolation and quantitative real-time RT-PCR analysis for miRNA-126 levels

Micro-RNAs were isolated from 50 mg of pulverized frozen xenograft tumor tissue using the miRNeasy Mini Kit (Qiagen, Maryland USA) following the manufacturer's recommended protocol. Reverse transcription (RT) was performed using the miScript II Reverse Transcription Kit (Qiagen, Maryland USA) with the following conditions: Two μg of total RNA were reverse transcribed in HiSpec Buffer, which facilitates selective conversion of mature miRNAs into cDNA. Twenty μL reactions were set up following manufacturer's specifications and incubated at 37°C for 1 h, then 95°C for 5 min. Real-time quantitative reverse transcriptase-polymerase chain reaction (qRT-PCR) for detection of mature miRNA-126 was performed using the miScript SYBR Green PCR Kit (Qiagen, Maryland USA). Cycling was done in a StepOnePlus machine (Applied Biosystems, California USA) with the following thermal profile: 95°C for 15 min, 40 cycles of 94°C for 15 s, 55°C for 30 s, 72°C for 30 s with optics on; flowed by a melt curve analysis. miRNA-126 sequences for rat and human are identical (miRBase.org).

#### Western Blot Analysis of KRAS protein levels as a downstream target of delivered miRNA-126

Cytosolic proteins were isolated from an aliquot of frozen pulverized xenograft tumors used for miRNA- 126 real-time qRT-PCR analysis. Western blot analysis was done as described 63 using equal amounts of cytosolic protein extract (30 µg) isolated from MDA-MB-231 and Panc1 CSC-derived xenograft tumors from test and control tumors. Cytosolic extracts were prepared by tissue homogenization in buffer containing 20 mM Tris-HCl pH 7.4, 1 mM EDTA, 250 mM sucrose, protease inhibitors, followed by centrifugation (11000g x 10 min) and final collection of supernatant (cytosolic fraction). Proteins were separated on a 15% SDS-PAGE and were transferred to PVDF membrane (Bio-Rad). The Western blot was reacted sequentially with anti-KRAS (Abcam cat# ab55391 at 20 µg/mL) followed by anti-b-actin (Santa Cruz cat# sc-47778 at 1 µg/mL) antibodies. Immunoreactive proteins were detected by chemiluminescence using the ECL Western Detection kit (GE Healthcare).

#### Comparative 9.4T MR-imaging of jNPs and precursor-USPIONs in phantoms

Comparative 9.4Tesla MR-imaging of jNP, precursor-USPIONs and blank control phantoms in 1% agar was conducted using gradient-echo at varying echo times (TE). Three 5 mm NMR tubes were filled with agar alone, or with agar containing jNPs or USPION-PEG-NH_2_ at a concentration of 0.5 x 10^12^ jNPs/mL in 1% agar. These tubes were then embedded in 2% agar inside a 26 mm diameter plastic tube, with their long axes parallel to the long axis of the outer tube. This assembly was placed at the isocenter of a 9.4T horizontal axis MRI scanner (Bruker Biospec, Billerica, MA) with the tubes oriented parallel to the magnetic field. Axial and coronal T2-weighted rapid acquisition with relaxation enhancement (RARE) images were acquired with TR/TE=3300/22 ms, echo train length = 4.

T2* relaxation times in the three vials were assessed using a multi-gradient echo sequence with TR=300ms, 26º flip angle, and 20 echoes per image acquired at echo times ranging from 4.08 to 99.08 ms in 5 ms steps. To minimize effects of macroscopic magnetic field variations (which can shorten T2*), two steps were taken. First, prior to each measurement, the magnetic field was shimmed locally using a point-resolved spectroscopy (PRESS) sequence to minimize the proton linewidth in a 5-6 mm cube centered on the vial of interest. The line width (FWHM) ranged from 7-13 Hz in the three vials prior to T2* measurement in each vial. Second, each multi-gradient echo image series was acquired with two voxel sizes. A first acquisition was performed with 1mm slice thickness and 0.312 mm in-plane resolution, and a second was then acquired with 0.5mm slice and 0.156 mm in-plane resolution. The second acquisition had half the voxel size of the first, and hence should have reduced intravoxel dephasing due to macroscopic magnetic field inhomogeneities. Under these conditions, the T2* measurements are consistent (within error) between the two data sets, with the discrepancies between the T2* values ranging from 1.0 to 1.7 times the standard error of the mean for the difference between the low- and high-resolution T2* values. T2* was determined by defining a circular region of interest covering 60 pixels within each vial in the lower-resolution data set, and independently fitting each pixel to a Gaussian function using a nonlinear least-squares algorithm (Mathematica, Wolfram Research, Champaign IL). Although the fits were performed using magnitude images, the inclusion of an offset to account for the noise background had a negligible (< 0.2 ms) impact on the fitted T2* values.

#### *Ex vivo* 9.4T MR-imaging of pancreatic peritoneal metastatic tumors

*Ex vivo* MR-imaging was performed on isolate tumors to ascertain no movement artifacts from breathing. Tumors were isolated 24 h after intravenous infusion of jNPs at 10^12^ jNPs/mL blood volume into xenografted nude rats with palpable pancreatic peritoneal tumors. We used non-targeted IgG-jNPs to assess universal tumor localization via enhanced permeability and retention (EPR). MR-images were acquired using a 3D gradient echo pulse sequence with TR/TE=25.3/6.5 ms and 31.8/13 ms, and with 30º flip angle. In-plane resolution was 200 μm, with 500 μm resolution in the slice-encoding direction. Regions containing high concentrations of jNPs would be detected by presence of hypointense (dark) signals at TE=6.5 ms. To confirm jNPs as cause for hypointensity, 9.4T MR-imaging was also done at TE = 13 ms which would increase the hypointensity if jNPs are in the region of interest in the tumors. Absence of jNPs or USPIONs would not show further signal dropout at longer TE=13 ms.

#### MR-imaging analysis of tumors and normal liver and kidney

*Ex vivo* MRI was performed in an 11.7 Tesla vertical-bore Bruker Avance spectrometer. We used the susceptibility weighted imaging (SWI) to analyze normal liver and kidney to increase sensitivity to detect jNPs in normal non-leaky microvasculature. SWI is a clinical MRI sequence that combines the phase and magnitude information and demonstrated to show sensitivity to iron particles as would be detected in venous blood, hemorrhage, iron storage or nanoparticles. For MRI of liver and kidney, the Fast Low Angle SHot (FLASH) clinical MRI sequence was used.

### Analysis of circulating cytokine levels after jNP infusion *in vivo*

Safety studies were done in Dahl salt-sensitive hypertensive rats using 10^12^ and 10^13^ jNPs/rat infused via tail vein without microbubbles compared to age-matched controls. Daily health monitoring was performed, and analysis of serum cytokine levels was done 2-weeks later by ELISA following manufacturer's specifications (Rat Cytokine/Chemokine Array, RayBiotech, GA). ELISA was done in duplicates per cytokine per rat, with 2 rats/group for 3 study groups.

### Statistical analysis

Sample sizes were chosen to meet power 0.8, alpha = 0.05, and differences in groups attaining *P* < 0.05 in parametric and non-parametric statistical tests. Data are presented as mean ± s.d. Where relevant, all data were checked for normality, and the appropriate statistical test applied for 3 groups: parametric 1-way ANOVA followed by Tukey multiple comparisons testing for data that pass normality testing, and non-parametric Kruskall- Wallis ANOVA with multiple comparisons by Dunns method for data that fail normality testing. Students t-test (normality passed) and non-parametric Wilcoxon rank sum test were used accordingly for differences between two groups. Chi square analysis was performed for categorical contingency group analysis. Two-way ANOVA repeated measures was used for analysis of MR-T2*-relaxivity (nanoparticle type x echo time) and for *in vivo* impact of jNPs on circulating cytokine profiles after 2 weeks (jNP-dose x cytokine type). GraphPad PRISM (Release 9.4, GraphPad, CA) software was used for statistical analyses.

## Supplementary Material

Supplementary figures.
