# Supplementary material for: Janus USPION modular platform (JUMP) for theranostic ultrasound-mediated targeted intratumoral microvascular imaging and DNA/miRNA delivery
Source: Theranostics. 2022 Nov 7;12(18):7646–67. doi: 10.7150/thno.78454 (PMC9706579; doi:10.7150/thno.78454)
Supplement: Supplementary file 1 — Supplementary figures. [file thnov12p7646s1.pdf]

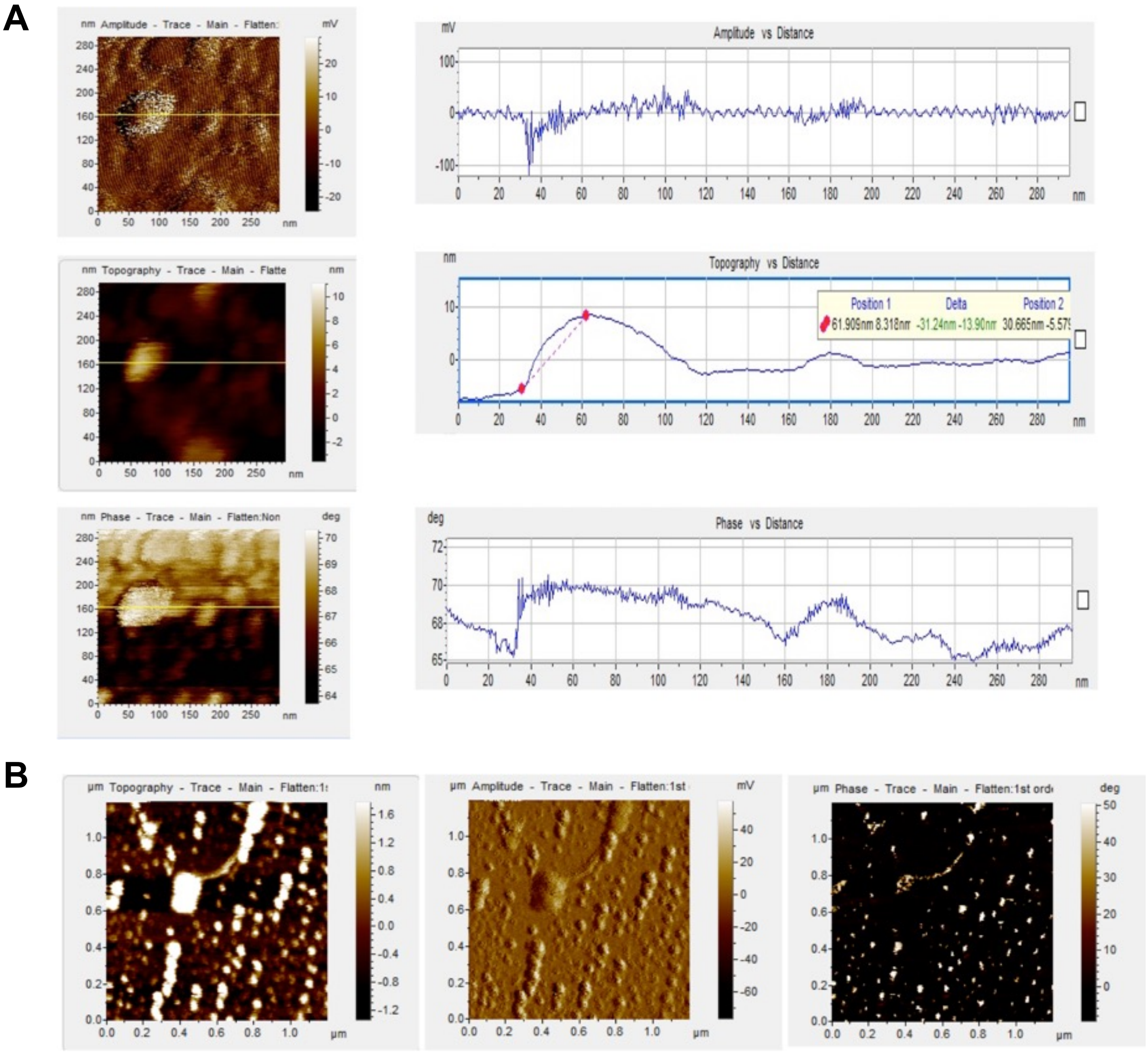

Figure S1. Representative Atomic Force Microscopy (AFM) images showing (A) analysis of an individual jNP showing amplitude, topography, and phase images with respective trace plots and (B) jNP binding to deposited lambda phage DNA on a mica surface.

(**A**) AFM amplitude, topography and phase images show the asymmetric avocado-like morphology of the jNP. The shortest radius, as calculated from the center of the iron oxide core to the closest edge, is 11.24 nm. All scaling of AFM images must account for the 20 nm radius tip. (**B**) AFM image of deposited lambda-phage DNA strand shows typical Y-shaped DNA-strand with jNPs attached to DNA-strand. The phase diagram (far right) highlights the difference of the iron oxide cores and the DNA strand from the mica surface.

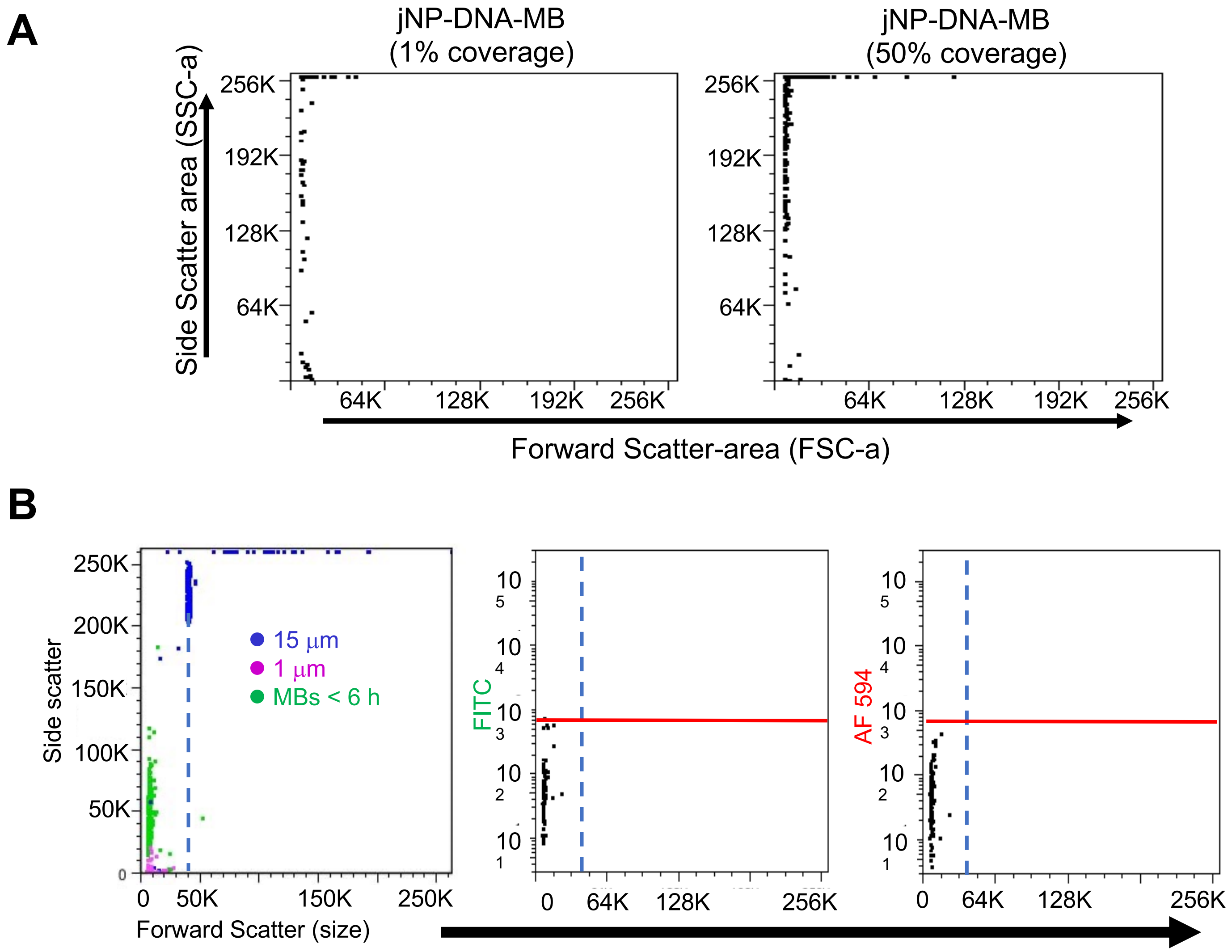

Figure S2. Additional *in vitro* characterization of jNPs: binding to DNA and to MBs-DNA.

(A) Representative flow cytometry image of side scatter area (SSC-a) detects increased granularity imparted by jNPs to MBs upon assembly of jNP-DNA-MBs. Y-axis, side scatter-area (SSC-a), corresponds to granularity; X-axis, forward scatter area, corresponds to size. Increased granularity is detected in jNP-DNA-MBs with increased jNP-coverage of MBs: 50% (~ 5 x 10^4/MB) vs 1% (10^3 jNPs/MB) coverage of MBs-DNA. (**B**) Flow cytometry analysis of different MB stocks (< 6 h from suspension) with 'bead markers' indicating 1-2 µm diameter MBs; equidistant 15 µm size marked by blue dash line; no autofluorescence of MBs detected for fluorophores used for DNA (AF488) and jNPs (AF594).

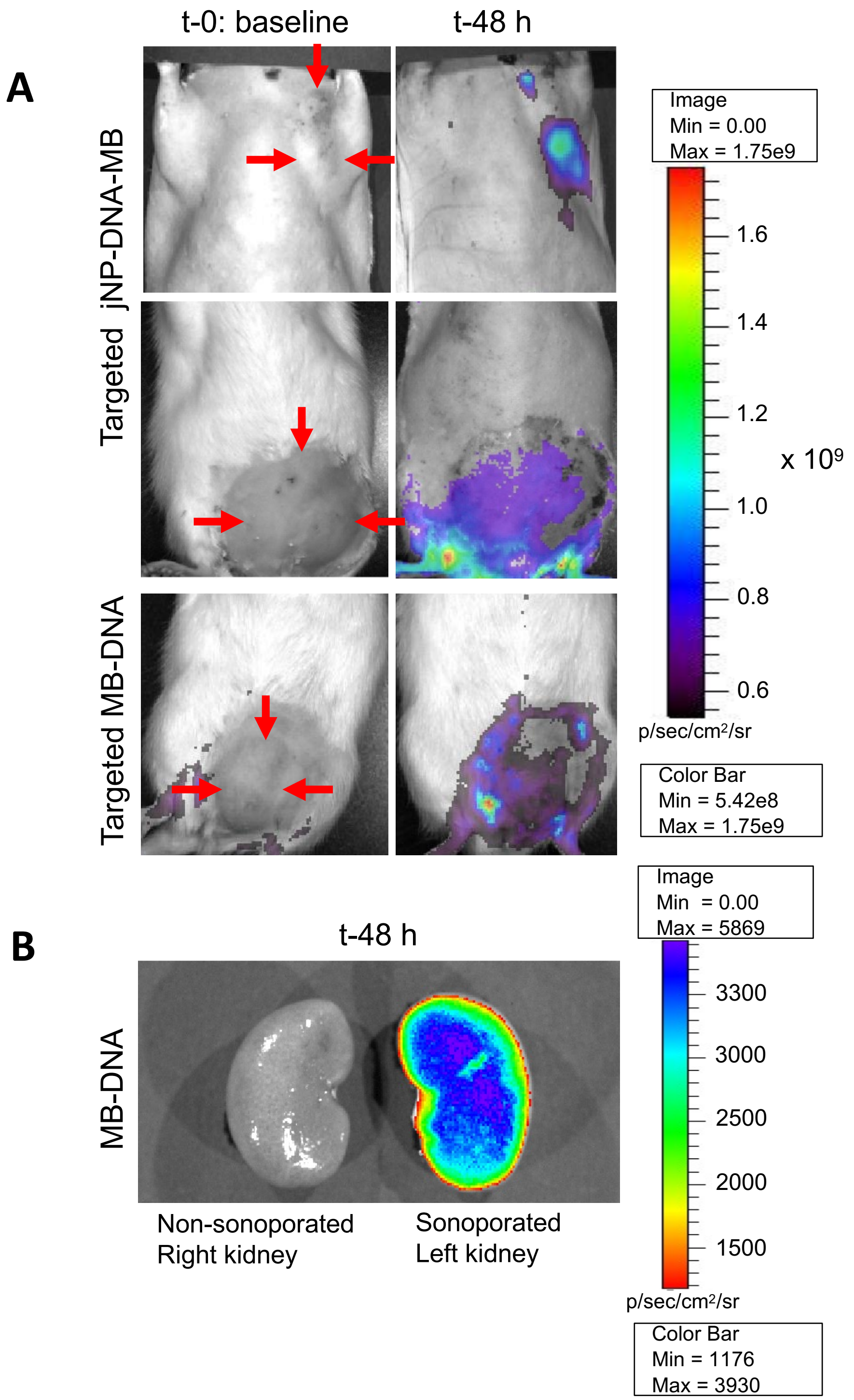

Figure S3. Comparative analysis of *in vivo* fluorescence imaging by IVIS. (**A**) Representative *in vivo* fluorescence images by IVIS at baseline and 48-hours after sonoporation. Zero-fluorescence shown at baseline prior to delivery of red fluorescent protein (RFP) minigene-DNA by sonoporation using targeted jNP-DNA-MBs and targeted MBs-DNA. Spontaneous mammary tumor in post-menopausal rat is designated by red arrows. Color bar scale: 5.42e8 to 1.7 e9; red = max; purple = min. (**B**) Comparative *in vivo* fluorescence images obtained by IVIS-imaging 48 h after sonoporation. Sonoporation was done using MBs-DNA immediately after infusion while

MBs filled the intravascular space (< 5 min) using a SoniGene sonoporator. Identical number of MBs and amount of RFP-DNA, and sonoporation settings were used. Color bar scale: 1176 to 3930; blue = max; red = min

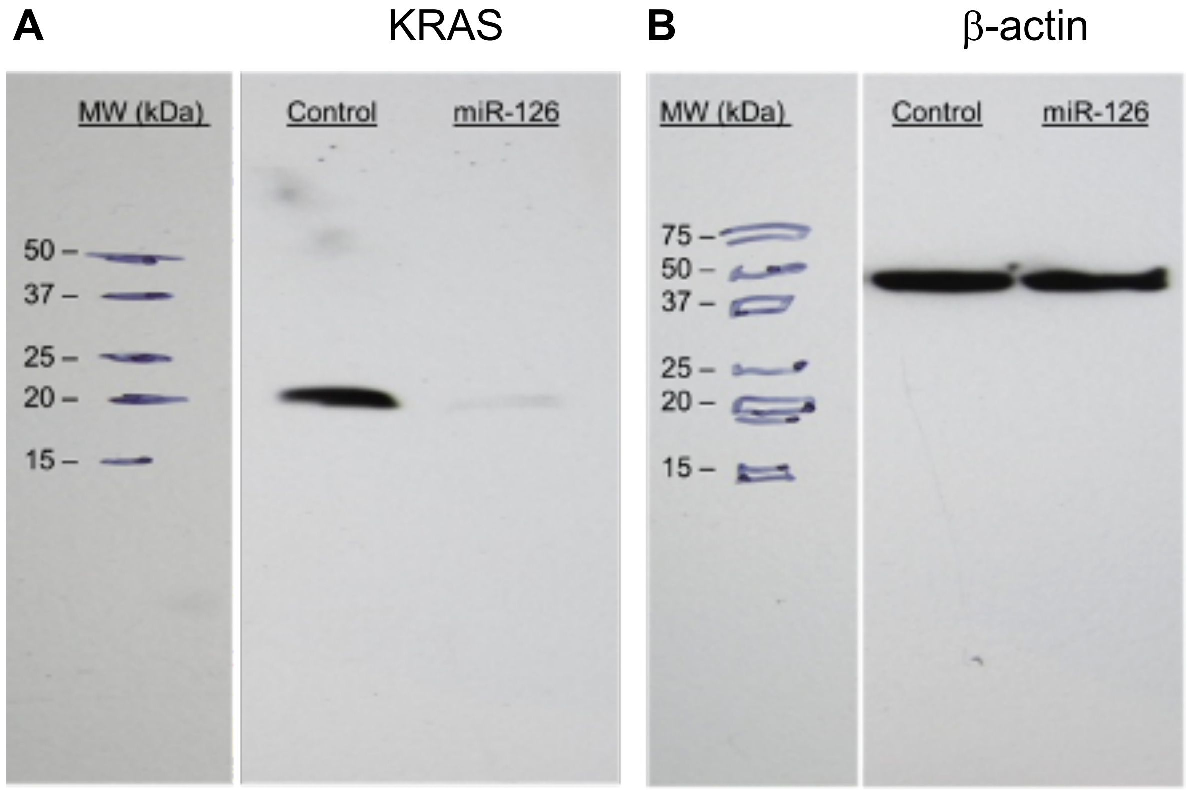

Figure S4. Full gel-image of Western blot analysis of KRAS and β-actin shown in Figure 6D. (**A**) Representative full gel-image of Panc1-tumor protein blot analysis of mir-126 target gene KRAS (22 kDa). (**B**) Full gel-image of Panc1-tumor protein blot analysis of internal control, β-actin (43 kDa). Molecular weight (mw) markers in kilodaltons (kDa) marked. Left lane, Control non-treated tumor, Right lane, miR-126, jNP-miR126-MB sonoporated tumor.

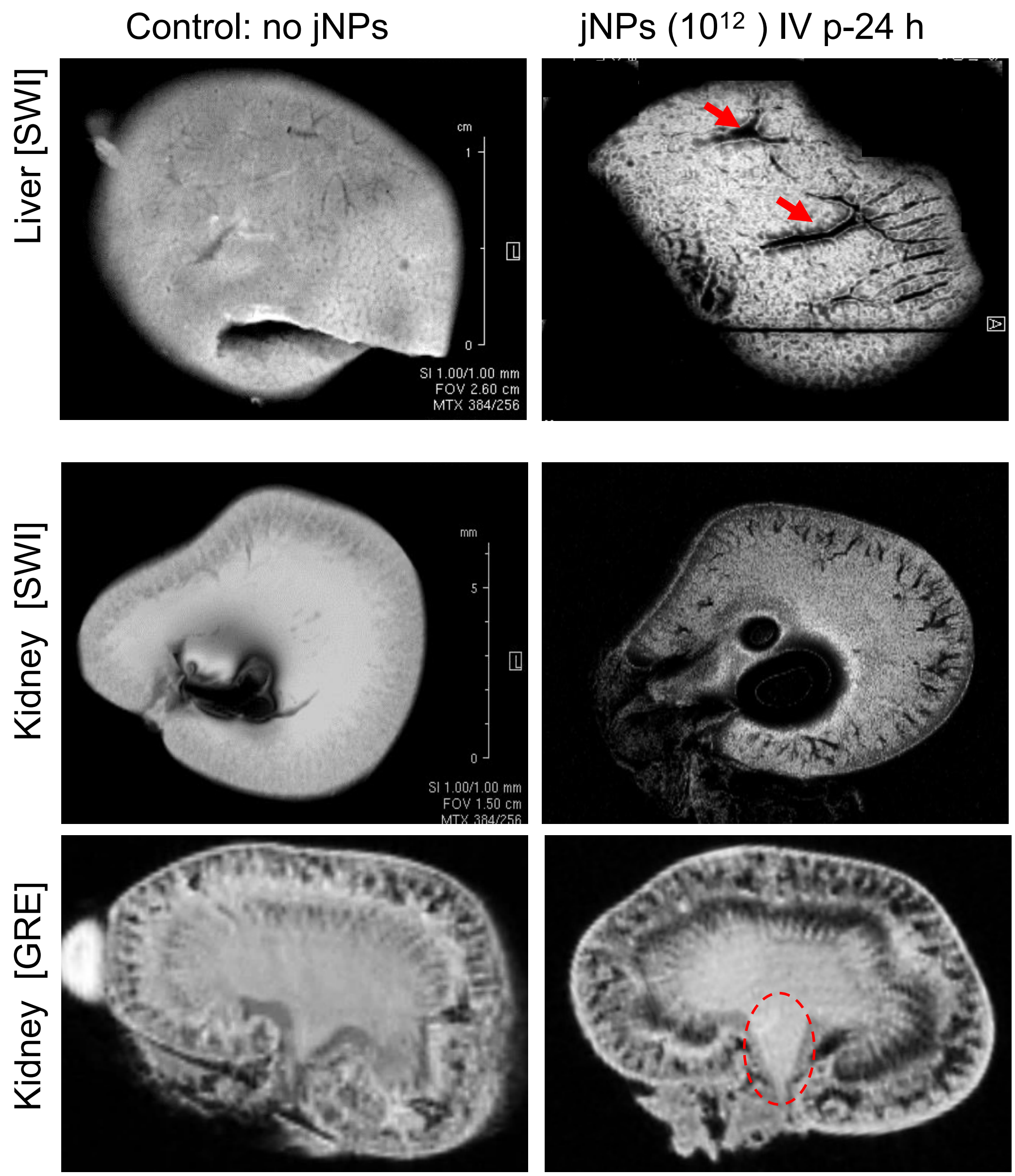

Figure S5. Representative MR-imaging of liver and kidney comparing control rats (no jNPs infused) and test rats (10^{12} jNPs/rat or 5 ng Fe/rat, ≥250 g rat) 24 h after IV infusion.

Ex vivo 11.7T MR-susceptibility weighted images (SWI) of liver and kidney from xenograft tumor rats show differential hypointensities in the liver and kidney consistent with defined anatomical structures rather than confluent areas of hypointensities seen in xenograft tumors (**Figure 5B**). *Ex vivo* 9.4T gradient echo (GRE) MR-images of control and test normal rat kidneys confirms MR-T2*-hypointensities in jNP-infused rat kidney, but not in the renal pelvis. Comparison of liver and kidney MR-images suggest hepatic clearance but not kidney excretion. MR-images were obtained with organs placed in fomblin. Histological correlates remain to be studied. Red arrows: hepatic ducts highlighted by USPIONs in jNPs consistent with hepatic excretion; Red dashed circle: kidney pelvis urine not highlighted by USPIONs in jNPs consistent with non-renal excretion.
